# Mrs4 loss of function in fungi during adaptation to the cystic fibrosis lung

**DOI:** 10.1128/mbio.01171-23

**Published:** 2023-07-11

**Authors:** Daniel Murante, Elora G. Demers, Tania Kurbessoian, Marina Ruzic, Alix Ashare, Jason E. Stajich, Deborah A. Hogan

**Affiliations:** 1 Department of Microbiology and Immunology, Geisel School of Medicine at Dartmouth, Hanover, New Hampshire, USA; 2 Department of Microbiology & Plant Pathology and Institute for Integrative Genome Biology, University of California-Riverside, Riverside, California, USA; 3 Department of Medicine, Dartmouth Health, Lebanon, New Hampshire, USA; Duke University Hospital, Durham, North Carolina, USA

**Keywords:** cystic fibrosis, fungi, iron, Mrs4, *Clavispora lusitaniae*, *Exophiala dermatitidis*, *Candida albicans*

## Abstract

The genetic disease cystic fibrosis (CF) frequently leads to chronic lung infections by bacteria and fungi. We identified three individuals with CF with persistent lung infections dominated by *Clavispora* (*Candida*) *lusitaniae*. Whole-genome sequencing analysis of multiple isolates from each infection found evidence for selection for mutants in the gene *MRS4* in all three distinct lung-associated populations. In each population, we found one or two unfixed, non-synonymous mutations in *MRS4* relative to the reference allele found in multiple environmental and clinical isolates including the type strain. Genetic and phenotypic analyses found that all evolved alleles led to loss of function (LOF) of Mrs4, a mitochondrial iron transporter. RNA-seq analyses found that Mrs4 variants with decreased activity led to increased expression of genes involved in iron acquisition mechanisms in both low iron and replete iron conditions. Furthermore, surface iron reductase activity and intracellular iron were much higher in strains with Mrs4 LOF variants. Parallel studies found that a subpopulation of a CF-associated *Exophiala dermatitidis* infection also had a non-synonymous LOF mutation in *MRS4*. Together, these data suggest that *MRS4* mutations may be beneficial during chronic CF lung infections in diverse fungi, perhaps, for the purposes of adaptation to an iron-restricted environment with chronic infections.

**IMPORTANCE:**

The identification of *MRS4* mutations in *Clavispora* (*Candida*) *lusitaniae* and *Exophiala dermatitidis* in individuals with cystic fibrosis (CF) highlights a possible adaptive mechanism for fungi during chronic CF lung infections. The findings of this study suggest that loss of function of the mitochondrial iron transporter Mrs4 can lead to increased activity of iron acquisition mechanisms, which may be advantageous for fungi in iron-restricted environments during chronic infections. This study provides valuable information for researchers working toward a better understanding of the pathogenesis of chronic lung infections and more effective therapies to treat them.

## INTRODUCTION

Evolution of pathogens in infections can lead to the rise of isolates with increased resistance to host defenses or drugs, improved fitness, or enhanced access to nutrients. An understanding of pathoadaptive mutations may improve therapies and treatments. The repeated rise of specific mutations in bacteria and fungi associated with chronic infections has been particularly well-documented in the context of lung infections in people with cystic fibrosis (CF). The genetic mutations that cause CF lead to chronic infections. Several studies have shown that nutritional immunity, mediated by innate immune effectors such as calprotectin which restrict access to metals, forces microbes to employ diverse strategies to acquire essential nutrients such as iron and zinc (1 - 3).

Many of the mutations that repeatedly arise in CF-related bacterial and fungal pathogens are in regulators (e.g., *lasR* and *mucA* in *Pseudomonas aeruginosa* and *agr* in *Staphylococcus aureus*). Analysis of *Candida* CF isolates has found similar regulatory mutations. A study of *Candida albicans* CF infection identified six instances of loss-of-function (LOF) mutations in *NRG1*, which encodes a repressor of filamentation; *nrg1* LOF mutants are resistant to the suppression of filamentation by the frequently co-infecting bacterium *Pseudomonas aeruginosa* (4). We previously published that a single *Clavispora* (*Candida*) *lusitaniae* infection, with no detectable co-infecting bacteria, had numerous activating and subsequent suppressing mutations in *MRR1* (5) that led to heterogenous resistance to the antifungal fluconazole, the toxic metabolite methylglyoxal, and *P. aeruginosa* toxins. Longitudinal collections of *Aspergillus fumigatus* isolates from a single individual with CF showed acquired mutations that led to high-osmolarity glycerol (HOG) pathway hyperactivation and improved fitness in the presence of oxidative and osmotic stress (6).

In this work, we describe a locus in *C. lusitaniae* (7) that was independently mutated in three separate subjects with CF. *C. lusitaniae*, a haploid member of the CTG clade within the Saccharomycetaceae family, is known to readily develop amphotericin B resistance (8), and can develop resistance to caspofungin and azoles (9, 10). *C. lusitaniae* has been reported in association with plant and food products, and it is less commonly found to be an abundant member of microbiome communities. Notably, *C. lusitaniae* is closely related to *Candida auris* which is also known for the repeated development of multidrug resistance, and is a critical threat on the WHO priority pathogens list (11, 12).

*C. lusitaniae* evolution in the CF lung may provide an opportunity to study fungal adaptation to host environments. We found non-synonymous mutations in *MRS4* arose independently in three different CF lung infections. Mrs4 is a high-affinity iron transporter that brings iron across the inner mitochondrial membrane into the inner lumen for processes including the synthesis of iron-sulfur clusters for incorporation into diverse enzymes and regulators. In other fungi, Mrs4-mediated iron transport is necessary for robust growth on medium with low iron and resistance to reactive oxygen species and menadione (13 - 18). Previous studies found that *MRS4* deletion significantly reduces virulence of *C. albicans* in a murine model for systemic candidiasis (19). We found that each of the Mrs4 variants had decreased function. RNA-seq analysis of isolates with different *MRS4* alleles in yeast extract peptone dextrose media (YPD) with and without iron chelator demonstrated that Mrs4 LOF led to significant increases in expression of multiple iron uptake pathways. Furthermore, strains with *MRS4* LOF alleles demonstrated increased accumulation of intracellular iron. A LOF mutation in *MRS4* was also found in CF infection isolates of *Exophiala dermatitidis*. Taken together, these data highlight that in two distinct environmental fungi, chronic infection leads to the selection for Mrs4 variants with decreased function and an increased capacity for iron uptake. Future studies will determine if these host-adapted strains have new vulnerabilities that can be exploited therapeutically.

## RESULTS

### *MRS4* mutations are found in *C. lusitaniae* from CF lung infections

**Fig 1:**
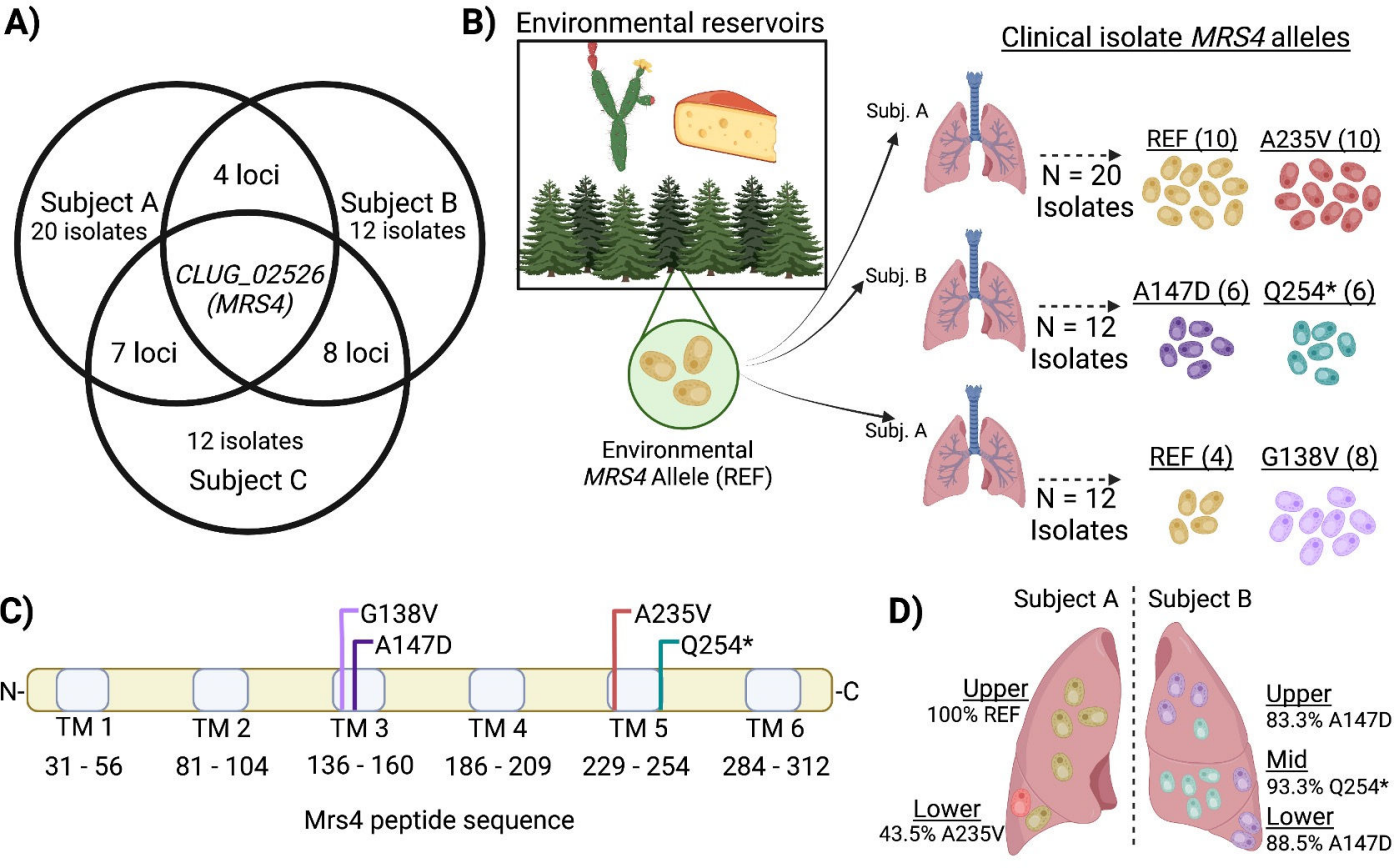
Non-synonymous SNPs in *MRS4* were found in whole-genome sequence data from 12 to 20 *C. lusitaniae* isolates from each of three subjects with chronic CF infections. (A) Analysis of loci that were heterogeneous in three *C. lusitaniae* populations in three separate individuals (subjects A, B, and C) found that only *CLUG_02526* (*MRS4*) had subpopulations with non-synonymous substitutions in all three infections. Subjects A, B, and C had 139, 59, and 207 variable loci within each respective isolate population. (B) Two *MRS4* alleles were detected in each population. “REF” indicates the *MRS4* sequence in environmental and acute infection isolates of *C. lusitaniae*. (C) The *MRS4* sequence encodes a barrel-structure iron transporter on the inner mitochondrial membrane; the protein is 318 amino acids long and comprised six transmembrane (TM) domains denoted by light blue bars. Each mutation is predicted to disrupt or truncate one of these transmembrane domains (see SuSPECT analysis, Fig. S3). (D) Pooled sequencing was performed on isolates from bronchoalveolar lavage fluid taken from specific lobes of subjects A and B. The relative abundances of *MRS4* alleles were quantified by analysis of individual reads.

Analysis of the microbiota in bronchoalveolar lavage (BAL) fluids collected at Dartmouth Health found three subjects with CF with lung infections dominated by *C. lusitaniae* [(7) and in unpublished data (E. G. Demers]. We sequenced the genomes of 12–20 isolates from each population (see Materials and Methods for accession numbers). To identify mutations that likely arose during infection, non-synonymous single nucleotide polymorphisms (SNPs) that were not fixed within the population from each individual were determined (Table S1). Nineteen genes had two alleles with non-synonymous differences in more than one population, defined as isolates from a single subject at a single time point, and only one gene had alleles with non-synonymous differences within all three populations: *CLUG_02526* (Fig. 1A). *CLUG_02526* encodes an amino acid sequence with 71% identity with *C. albicans* SC5314 Mrs4 (19) and 51% with *Saccharomyces cerevisiae* S288C Mrs3 and Mrs4 (Fig. S1). Mrs4 functions as high-affinity mitochondrial iron importer in both species (16, 20). Due to the high percentage of sequence identity and our phenotypic characterization of *CLUG_02526* deletion mutants (described below), we will heretofore refer to *CLUG_02526* as *MRS4*.

To compare the *MRS4* sequences from CF *C. lusitaniae* isolates to *MRS4* sequences in a broader collection of *C. lusitaniae* strains, we also analyzed *MRS4* in 10 *C. lusitaniae* isolates from diverse clinical and environmental sources. We found that the predicted Mrs4 amino acid sequences were identical and the genes differed by only a small number of synonymous SNPS that varied among alleles (Fig. S2). The conserved Mrs4 amino acid sequence will be referred to as the “reference” or Mrs4^REF^ sequence. Isolates with *MRS4* alleles that encoded the Mrs4^REF^ sequence were found in both subject A and subject C (Fig. 1B). In addition to the reference allele, subject A and subject C populations each had isolates with mutant alleles that differed by single non-synonymous SNPs, and encoded *MRS4^A235V^* and *MRS4^G138V^*, respectively (Fig. 1B). Subject B isolates carried one of two mutant alleles that encoded *MRS4^A147D^* and MRS4^Q254*^ (Fig. 1B), suggesting that two independent *MRS4* mutant lineages arose within that population. The Mrs4 substitutions or terminations occurred in predicted transmembrane alpha helices of the protein (Fig. 1C) and occurred at residues that were conserved across diverse species (Fig. S1). Each *MRS4* mutation found in the CF *C. lusitaniae* isolates had a high likelihood of affecting function based on the SuSPECT analysis method which estimates the probability for single amino acid variants to impact phenotype (Fig. S3) (21).

### Spatial and longitudinal analyses show that *MRS4* alleles may have arisen independently in different regions of the lung and that Mrs4 LOF isolates persisted over time and across different lobes of the lung

Using whole-genome sequence data for pools of 75–96 isolates from the upper and lower lobes of the right lung of subject A and the upper, middle, and lower lobes of the left lung of subject B, we determined the fraction of reads that encoded the *MRS4* SNPs described above within each pool. For the pooled isolates of the upper lobe of subject A, the reads contained only the *MRS4^REF^* allele, while ~43% of sequenced population from the lower lobe had the *MRS4^A235V^* allele (Fig. 1D). Both alleles were detectable in the upper, middle, and lower lobe isolate pools of subject B. The *MRS4^A147D^* allele was present at a higher percentage in the upper and lower lobe (~83% and 89%, respectively), while MRS4^Q254*^ was found in ~93% of reads in the middle lobe. Sequence analysis of four sputum isolates from a sample donated by subject A, collected ~1 year after the BAL isolates were recovered, found two isolates with *MRS4^REF^* and two isolates with *MRS4^A235V^* suggesting that the Mrs4 variant-containing isolates persisted over time. Similarly, we obtained six respiratory sputum and stool isolates from subject B more than 1 year after the initial isolation and amplified and sequenced the *MRS4* allele. We found that they all contained the *MRS4^A147D^* allele. These longitudinal isolates indicate that the *MRS4* mutations persisted over time and possibly in multiple compartments, and thus were not transiently present only at the time of initial isolation from BAL fluid.

### *MRS4* variants confer LOF phenotypes

**Fig 2:**
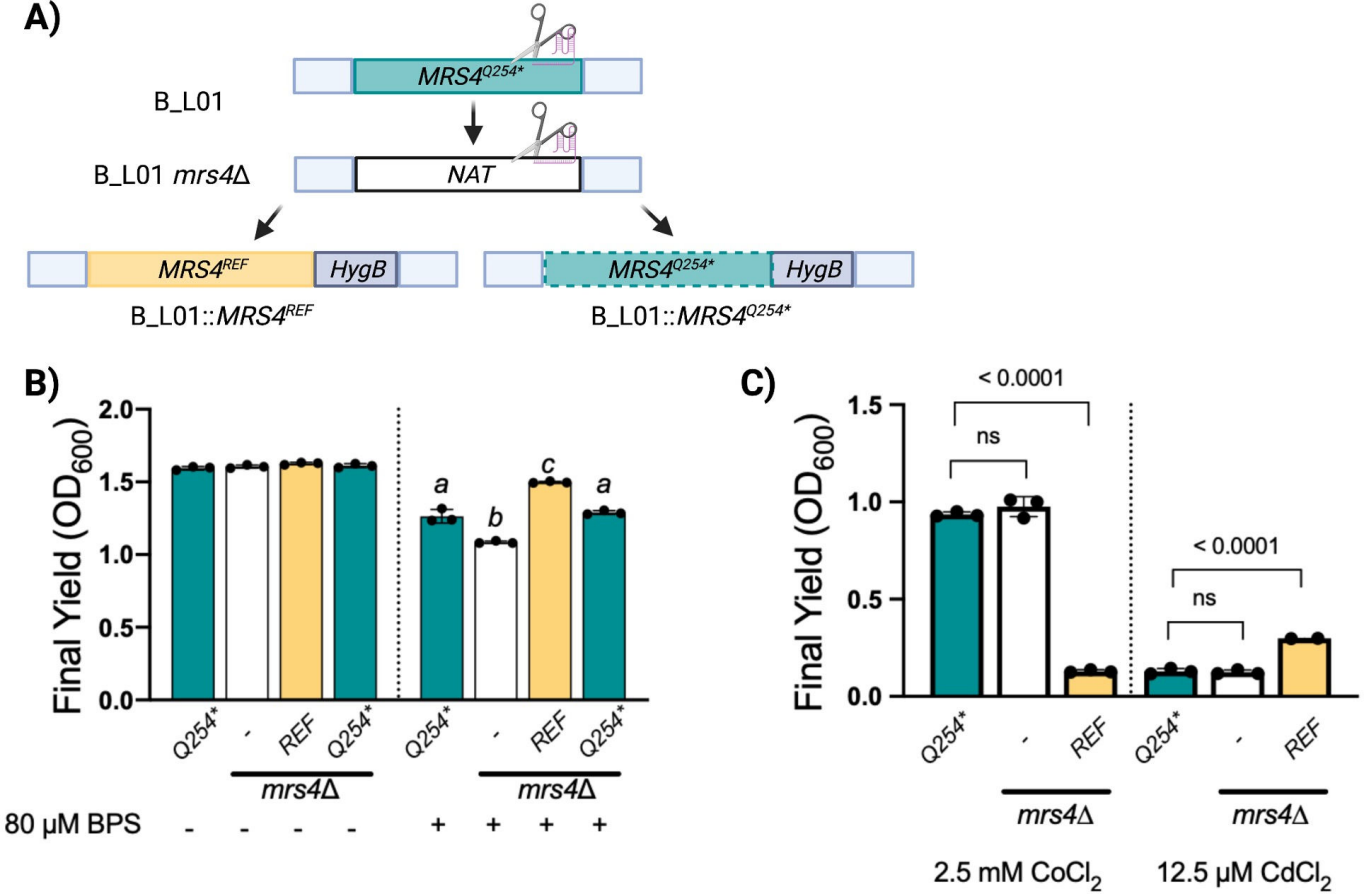
Mrs4^Q254*^ confers loss of function in *C. lusitaniae*. (A) An *mrs4*∆ mutant and *mrs4*∆ mutants complemented with MRS4^Q254*^ or the *MRS4^REF^* were constructed in the B_L01 clinical isolate background. (B) Strains were assessed for growth in a 96-well plate after 24 h at 37°C in YPD or YPD with 80 µM BPS iron chelator. Columns labeled with *a* are non-significantly different from each other and are significantly different from columns labeled with *b* and *c*. (C) Indicated strains were grown for 24 h at 37°C in YPD supplemented with 2.5 mM CoCl_2_ (left) and 12.5 µM CdCl_2_ (right). There were at least three replicates per sample. Indicated *P*-values are from a one-way analysis of variance (ANOVA) with Tukey’s *post hoc* correction. ns, not significant.

In other fungi, the deletion of *MRS4* impairs growth in low iron media or the presence of an iron chelator (17, 20, 22). To determine the activity of Mrs4 variants in the *C. lusitaniae* CF isolates, we first constructed an *mrs4*∆ derivative of subject B isolate B_L01, which had an MRS4^Q254*^ allele, then complemented back either the native B_L01 MRS4^Q254*^ allele or *MRS4^REF^* at the native locus (Fig. 2A). We chose the *MRS4* allele from strain ATCC 42720 as the source of the *MRS4^REF^* sequence. We observed that all four isogenic strains (B_L01 parental isolate, the *mrs4*∆ mutant, and the *mrs4*∆ mutant complemented with *MRS4^REF^* or MRS4^Q254*^) reached similar yields in YPD (Fig. 2B). Yields were reduced in YPD amended with a high-affinity ferrous iron chelator bathophenanthroline disulfonate (BPS), and we observed differences dependent on *MRS4* allele (Fig. 2B). Deletion of *mrs4* in the B_L01 background reduced final yield. Complementation with the *MRS4^REF^* allele restored growth to a significantly greater degree than the parent strain, or to the B_L01 *mrs4*∆ complemented with the native allele. We also observed sensitivity to bathophenanthroline in a defined minimal medium of yeast nitrogen base, although less chelator was required to restrict growth in this condition (Fig. S4). These results indicate that the truncated variant has decreased function relative to the reference allele, but that the truncated variant may retain some function.

Deletion mutants lacking *MRS4* in *S. cerevisiae* and *C. albicans* have altered metal sensitivities such that mutants are more resistant to cobalt and more sensitive to cadmium when compared to their Mrs4+ counterparts (17, 20) due to activation of transcription factors such as Aft1 in *S. cerevisiae* (15). We found that cadmium and cobalt affected the growth of *C. lusitaniae* in an *MRS4* allele-dependent manner. The B_L01 *mrs4*∆ derivative and the *mrs4*∆ complemented with the allele encoding Mrs4^Q254*^ were more resistant to cobalt than the *mrs4*∆ mutant complemented with *MRS4^REF^* (Fig. 2C). Conversely, the *mrs4*∆ strain complemented with the Mrs4^REF^ variant was more resistant to cadmium than the *mrs4*∆ mutant or the mutant complemented with Mrs4^Q254*^. Together, these data further suggest that the Mrs4^Q254*^ variant is less functional than the reference allele.

**Fig 3:**
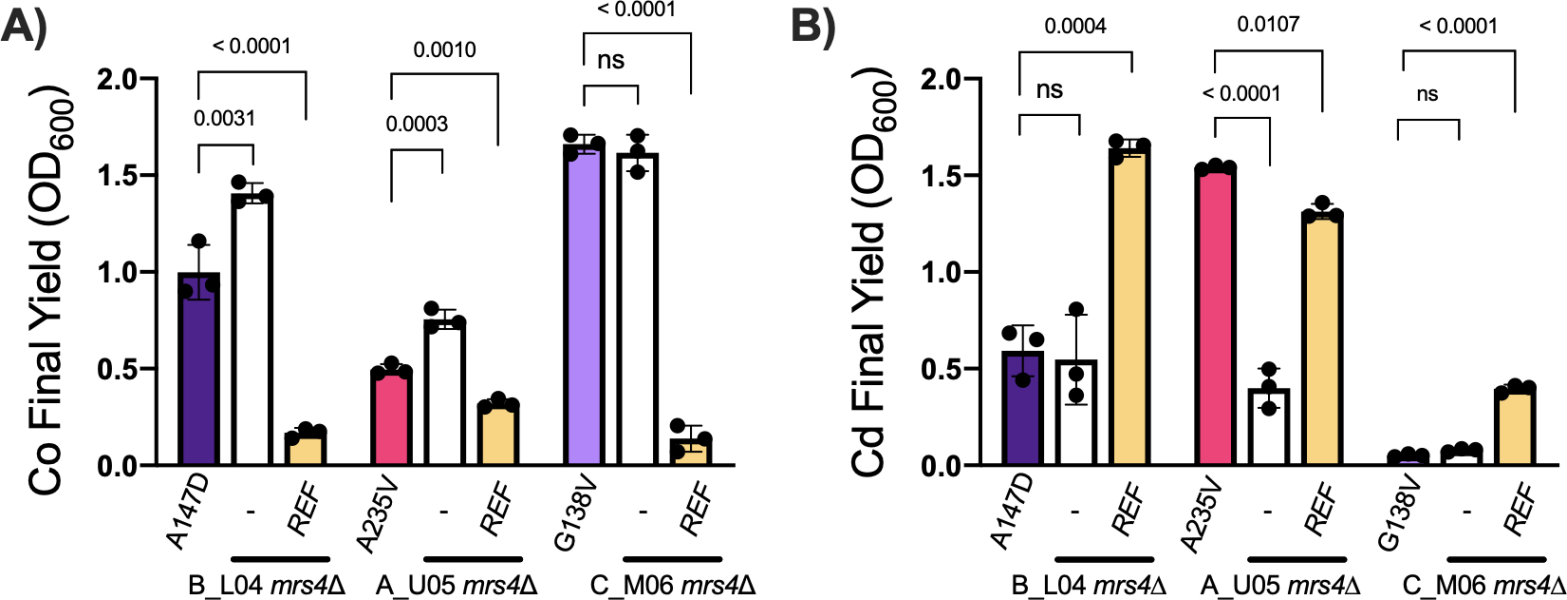
*MRS4* mutations in each clinical population demonstrate LOF phenotypes. Representative parent isolates of each mutation from subject B (B_L04, *MRS4^A147D^*), subject A (A_U05, *MRS4^A235V^*), and subject C, (C_M06, *MRS4^G138V^*) and their *mrs4*∆ derivatives that were then complemented with the *MRS4^REF^* allele were grown in YPD supplemented with (A) 2.5 mM CoCl_2_ and (B) 12.5 µM CdCl_2_. Data represent the endpoint OD600 measured by a Synergy Neo2 plate reader after 24 h of growth at 37°C. Indicated *P*-values are from one-way ANOVA with Tukey’s *post hoc* correction. ns, not significant.

To characterize the levels of function for the other three Mrs4 variants found in the CF clinical *C. lusitaniae* isolates, we created *mrs4*∆ derivatives of isolates A_U05 (*MRS4^A235V^*), B_L04 (*MRS4^A147D^*), and C_M06 (*MRS4^G138V^*), which were then complemented with the *MRS4^REF^* allele. In each case, complementation with the functional *MRS4^REF^* allele made cells more sensitive to cobalt (Fig. 3A) as was the case for strain B_L01. Similarly, replacement of *MRS4* with the *MRS4*^REF^ allele increased cadmium sensitivity in B_L04 (*MRS4^A147D^*) and C_M06 (*MRS4^G138V^*) (Fig. 3B). Unexpectedly, the A_U05 (*MRS4^A235V^*) isolate had greater resistance to cadmium than the same background with the *MRS4^REF^* allele, perhaps due to other genetic differences. When we expressed *MRS4^A235V^* in B_L01 *mrs4*∆, the resultant strain was more sensitive to cadmium than the isogenic strain with *MRS4^REF^* supporting the conclusion that the Mrs^A235V^ variant had low or no activity (Fig. S5). We also made an *mrs4*∆ mutation in an outgroup *C. lusitaniae* strain RSY284 (DH2383) (23) with a native *MRS4*^REF^ allele, and confirmed that the mutant had the expected resistance to cobalt and sensitivity to cadmium (Fig. S5A). Together, these data suggest that LOF mutations in *MRS4* arose four independent times across three chronic CF infections indicating possible selection for phenotypes associated with loss of Mrs4 function.

#### Characterization of *MRS4* growth phenotypes

Mitochondrial metabolism is highly dependent on the availability of metals such as iron. To assess whether the *MRS4* loss-of-function alleles affected mitochondrial activity, we examined the growth of isogenic strains with either *MRS4*^REF^ or Mrs4^Q254*^ in medium with either glucose (which can be fermented) or glycerol as the major carbon source. In both yeast extract peptone medium and yeast nitrogen base media with 2% glucose, there were no significant differences in growth for the strain with Mrs4^REF^ compared to the *mrs4* null mutant or strains with Mrs4^Q254*^ (Fig. S6A and C). Similar results were obtained when grown in medium with glycerol as the dominant or sole carbon source (Fig. S6B and D). Robust growth and lack of differences suggested that there was no major defect in respiratory metabolism.

As a more sensitive indicator of changes in levels of respiration and fermentation, we quantified glucose consumption relative to fermentation product production by high-performance liquid chromatography (HPLC). Analysis of supernatants from cultures of the *mrs4*∆ + *MRS4*^REF^ strain grown in medium with glucose as the sole carbon source found that the dominant fermentation product was acetate followed by ethanol; glycerol was not detected. Under the same conditions, *C. albicans* strain SC5314 produced mainly ethanol with low levels of acetate and glycerol. Normalized to moles of carbon, *C. lusitaniae* converted more than twice as much glucose to fermentation products than *C. albicans* (Table S2). When the mass balance of glucose consumed and fermentation products made for *C. lusitaniae mrs4*∆ + MRS4^Q254*^ and *mrs4*∆ + *MRS4*^REF^ were compared, we found no significant differences suggesting comparable levels of respiration and fermentation (Table S2). We also constructed a *C. albicans mrs4*∆/∆ homozygous mutant and found no significant difference in fraction of carbon used for fermentation when the SC5314 wild type was compared to the *mrs4*∆/∆ homozygous mutant (Table S2).

To further assess metabolic differences associated with Mrs4 LOF, we analyzed growth of B_L01 parent and *mrs4*∆*::MRS4^REF^* on different sole carbon sources using the Biolog carbon utilization phenotype microarray plates. We inoculated equal concentrations of each strain in YNB minimal medium and observed growth over the course of 48 h. Across the 192 carbon sources tested, there were no differences in growth that were confirmed in secondary analysis ( Supplementary dataset 1). Analysis of B_L01 parent and B_L01::*MRS4^REF^* did not show any differences in minimal inhibitory concentrations for commonly used antifungals including fluconazole and amphotericin B (data not shown). Consistent with published studies in *Cryptococcus neoformans* (24), in *C. lusitaniae* DH2383 and B_L01, functional Mrs4 was necessary for full H_2_O_2_ resistance (Fig. S6E and F).

### *MRS4* LOF impacts expression of iron homeostasis regulators and metal storage

**Fig 4:**
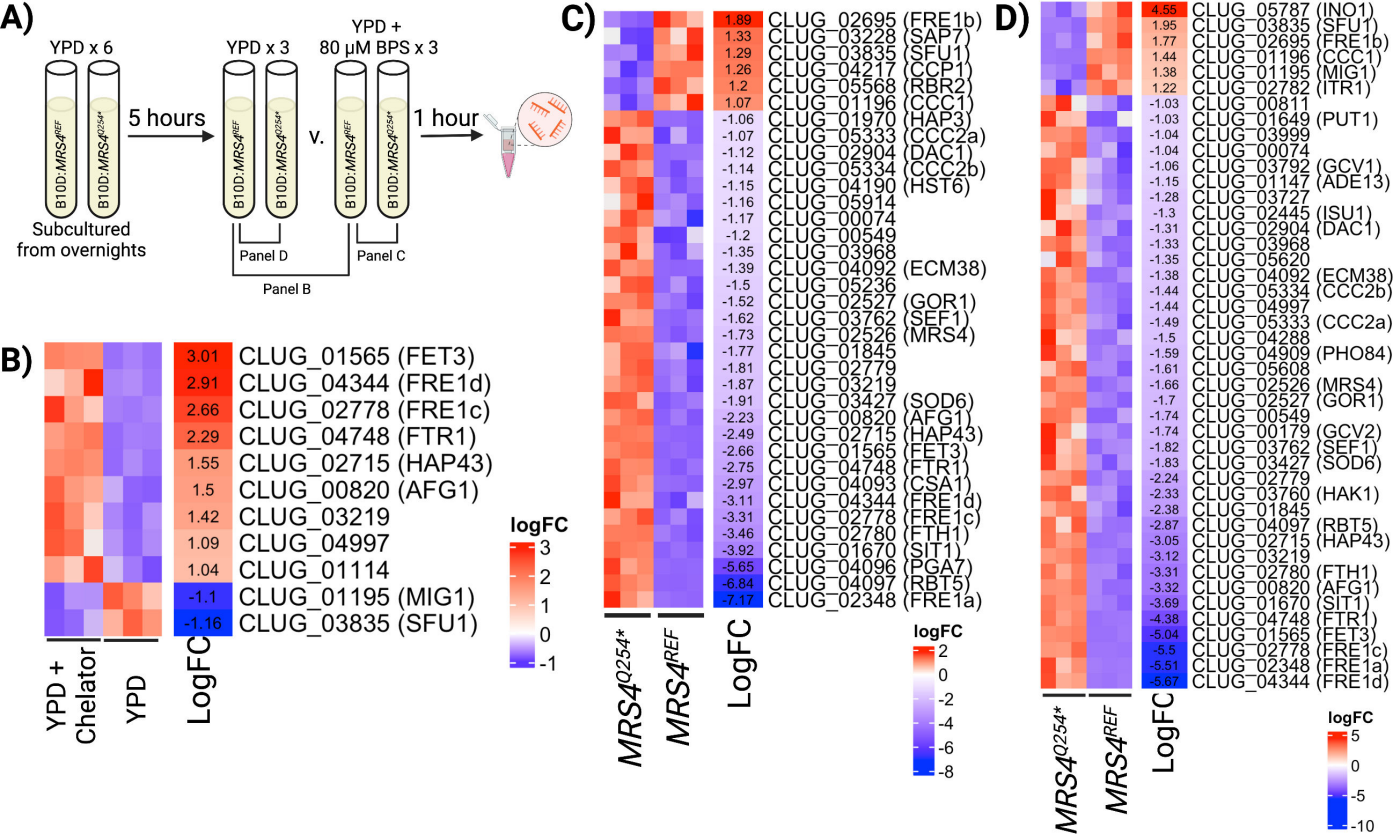
Loss of Mrs4 function leads to increased expression of iron acquisition genes. (A) Design of RNA-seq sample preparation. Sextuplicate cultures of B_L01 *mrs4*∆ complemented with either *REF* or Q254* MRS4 alleles were grown overnight, then sub-cultured into YPD and grown for 5 h. Cultures were grown for an additional hour with either 80 µM BPS or vehicle prior to RNA isolation. Gene expression heatmaps indicate differentially expressed genes (*P* < 0.05 and a log_2_ fold-change ≥|1|) in a comparison between (B) the B_L01::*MRS4^REF^* strain grown in YPD (iron replete) or YPD with BPS (iron deplete), (C) B_L01::*MRS4^REF^* and B_L01::MRS4^Q254*^ grown in YPD with BPS, and (D) B_L01::*MRS4^REF^* and B_L01::MRS4^Q254*^ grown in YPD. Gene names in parentheses indicate predicted orthologs in *C. albicans*. When more than one *C. lusitaniae* gene is predicted to be most similar to the same *C. albicans* gene (*FRE1* and *CCC2*), the alleles are differentiated with lowercase letters.

To gain insight into how Mrs4 LOF affected *C. lusitaniae*, we performed a transcriptomics analysis of B_L01 *mrs*∆ with *MRS4^REF^* and MRS4^Q254*^. Because cells with defective Mrs4 (e.g., MRS4^Q254*^) showed reduced growth on medium with chelator (Fig. 2B), we performed an RNA-seq analysis of cells from mid-exponential phase cultures growing in YPD and parallel cultures that received a 1-h exposure to the iron chelator BPS (Fig. 4A for experimental scheme). The short exposure to BPS was used to limit the pleiotropic effects associated with growth differences. Comparison of the strains with *MRS4^REF^* without or with exposure to iron chelator found 11 genes that had a fold difference greater than log_2_ 1 and a false discovery corrected *P*-value less than 0.05 (Fig. 4B; Supplementary dataset S2). Among the genes that were differentially expressed were the predicted orthologs of cell surface high-affinity ferric iron uptake genes in *C. albicans* (*FTR1*, *FRE1*, and *FET3*) and a gene that encodes a predicted regulator involved in iron utilization *SFU1* (Fig. 4B) which is known to be transcriptionally downregulated in response to iron limitation response in other *Candida* species (25, 26). Among the differentially expressed genes, there were four genes (CLUG_02348, CLUG_02695, CLUG_02778, CLUG_04344) that were most similar to *C. albicans FRE1* that we refer to as FRE1a-d, respectively, and two genes (CLUG_05333 and CLUG_05334) that were most similar to *C. albicans CCC2*, which we referred to as CCC2a and b, respectively.

Comparison of the transcriptomes of B_L01 *mrs*∆ + *MRS4*^REF^ to B_L01 *mrs4*∆ + MRS4^Q254*^ after chelator exposure found that ferric reductases that were induced upon exposure to chelator in the Mrs4^REF^ strain were higher in cells with Mrs4^Q254*^, as well as additional genes involved in ferric iron reduction *FTH1* and *FRE1* homolog FRE1a (Fig. 4C). We found differential expression of the *SFU1, SEF1*, and *HAP43* regulators and also found greater fold induction of the gene encoding pro-iron acquisition transcription factor *SEF1* in the *mrs4*∆ + MRS4^Q254*^ relative to *mrs*∆ + *MRS4*^REF^ with chelator. In addition, we found differential expression of putative orthologs of genes for alternative forms of iron uptake (*SIT1*, *CSA1, RBT5, PGA7*) and intracellular metal transporters for copper and iron localization (*CCC1*, CCC2a/b) (27), respectively, when Mrs4 function was low. *MRS4* transcript levels were log_2_ 1.7-fold higher in the *mrs4*∆ + MRS4^Q254^^*^ strain, and the *MRS4*-adjacent gene *GOR1*, predicted to encode a glyoxylate reductase, was also significantly higher in the Mrs4^Q254*^ bearing strain.

We also compared transcriptomes of *mrs*∆ + *MRS4*^REF^ and *mrs4*∆ + MRS4^Q254*^ in control conditions without chelator. Thirty-six genes were differentially expressed across the two strains in the presence of chelator (Fig. 4C), and 27 of them were still differentially expressed in its absence (Fig. 4D). Ferric reductase encoding genes and their regulators (e.g., *HAP43*) were again differentially expressed (Fig. 4C and D). An independent experiment, using reverse transcription quantitative real-time PCR (RT-qPCR) analysis of RNA from *mrs*∆ + *MRS4*^REF^ and *mrs4*∆ + MRS4^Q254*^, also found transcript levels of *HAP43* and *FTR1* to be significantly higher in both iron-replete and iron-chelated conditions (Fig. S7). We also observed that *mrs*∆ + *MRS4*^REF^ had ~4.5-fold higher levels of the predicted ortholog of inositol-3-phosphate synthase *INO1*, and 1.22-fold higher levels of the predicted ortholog of inositol transporter *ITR1* than *mrs4*∆ + MRS4^Q254*^ in YPD (Fig. 4D).

### Loss of Mrs4 function increases tetrazolium reduction, suggesting greater surface ferric reductase activity

**Fig 5:**
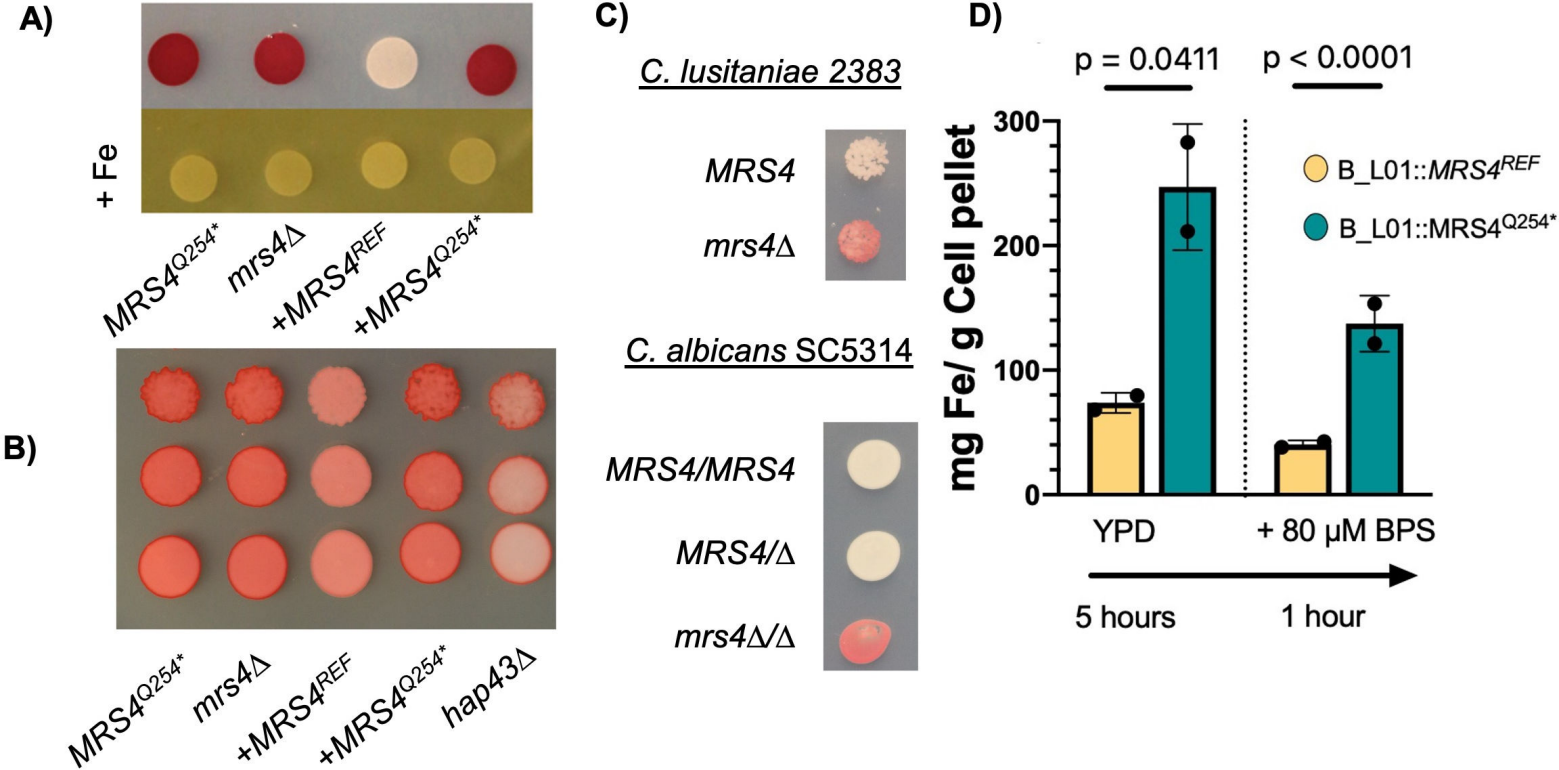
Decreased Mrs4 function increases ferric reductase activity and intracellular iron content. (A) B_L01-derived strains *mrs4*∆*, mrs4*∆*::MRS4^REF^*, and *mrs4*∆::MRS4^Q254*^ were spotted on YNB-glycerol plates. Plates were incubated for 24 h at 37°C. Each plate was overlayed with a 10 mL solution of 1 mg/mL tetrazolium chloride (TTC) and incubated for 5 min prior to imaging. Red pigmentation represents greater levels of ferric iron reduction. Inclusion of 10 mM of FeCl_3_ (+Fe) as a competitor eliminates TTC reduction. (B) B_L01-derived strains *mrs4*∆*, mrs4*∆*::MRS4^REF^, mrs4*∆::MRS4^Q254*^, and *hap43*∆ were serially diluted from 1 OD and spotted on YPD plates, then allowed to grow for 24 h at 37°C, and then analyzed as in panel A. (C) *C. lusitaniae* DH2383 and its *mrs4*∆ mutant and *C. albicans* SC5314 with single and double knockouts of *mrs4* were analyzed for surface ferric reductase activity on YNB-glycerol. (D) B_L01 *mrs4*∆ strains complemented with *MRS4^REF^* and MRS4^Q254*^ alleles were grown in YPD or YPD +80 µM BPS as outlined in Fig. 5A. Whole-cell iron was quantified using inductively coupled plasma mass spectrometry (ICP-MS). Data represent the averages of three technical replicates for two experiments done on separate days. Indicated *P*-values are from Student’s *t*-tests.

We sought to determine if the higher levels of in transcripts encoding cell surface ferric reductases in MRS4^Q254*^ strains, even in iron-replete conditions, translated into higher levels of iron acquisition activity. To do so, we utilized tetrazolium chloride (TTC), a substrate for ferric reductases (28). To avoid complications associated with growth inhibition by TTC, we grew colonies on YNB-glycerol agar for 24 h, then overlaid with molten 1% agar containing TTC (Fig. 5A). Upon TTC reduction by ferric reductases, an insoluble red pigment forms. After 10 min, there was a strong red coloration associated with colonies formed by MRS4^Q254^^*^ and the *mrs4*∆ strains, but not the *MRS4^REF^* strain (Fig. 5A). The TTC reduction phenotype was abrogated by the addition of excess ferric iron in the agar overlay (Fig. 5A). These data suggest greater ferric reductase activity on the cell surface when Mrs4 activity is low. Similar results, though to a lesser degree, were observed on YPD medium (Fig. 5B).

Previous studies in *C. albicans* have shown that deletion of *MRS4* leads to altered cell iron content and expression of iron regulon genes such as *AFT2,* which encodes a mitochondrial iron-responsive transcription factor (20, 29). However, as *C. lusitaniae* lacks an ortholog with significant similarity to Aft2, we hypothesized that CLUG_02715, the predicted ortholog of Hap43 [part of the Hap43-Sfu1-Sef1 iron-responsive transcription factor network (30)] was part of this response that led to increased expression of iron uptake genes. Thus, we analyzed a *hap43*∆ mutant in the B_L01 background with the defective Mrs4^Q254*^ variant, and found that Hap43 indeed contributed to the increased levels of iron reductase activity created by Mrs4 LOF (Fig. 5B). We also found that surface iron reductase activity was higher in the *C. lusitaniae* strain DH2383 *mrs4*∆ and *C. albicans* SC5314 *mrs4*∆/∆ relative to their respective parent strains. In *C. albicans*, both copies of *MRS4* needed to be deleted for manifestation of the increased tetrazolium reduction phenotype (Fig. 5C).

### Mrs4 LOF leads to the accumulation of intracellular iron

To evaluate the consequences of differential expression of iron acquisition genes in strains with and without Mrs4 function, we analyzed the concentrations of cellular iron by ICP-MS. In iron-replete YPD medium, the strain *mrs4*∆ + MRS4^Q254*^ had significantly higher levels of intracellular iron than *mrs4*∆::*MRS4^REF^*(Fig. 5D). In cells subjected to chelator treatment for 1 h prior to harvest, intracellular iron was lower than in the untreated cells, but still significantly higher in the *mrs4*∆::MRS4^Q254*^ strain. These data suggest that the observed increase in iron acquisition gene transcripts was concomitant with higher intracellular iron which may be advantageous *in vivo* and may explain the repeated LOF of Mrs4 in clinical *C. lusitaniae* populations.

### *MRS4* mutations in other fungi of clinical interest

In light of variation in *MRS4* in three different *C. lusitaniae* populations, we investigated the consequences of two other naturally occurring *MRS4* allelic variations that we observed in other species. First, we assessed the activity of Mrs4 variants in *Candida auris,* a fungus that emerged within the past 40 years (31) and is closely related to *C. lusitaniae*. Multidrug-resistant strains that caused localized outbreaks emerged independently within genetically distinct clades. We analyzed *Candida auris MRS4* alleles within and between clades, available from published sequences (32), and found that the encoded Mrs4 sequences were identical, with one exception. Clade I *MRS4* alleles encoded the Mrs4^31V^, while other clades encoded Mrs4^31A^. This difference was confirmed by Sanger sequencing at the Dartmouth Molecular Biology Core. To assess the activity of these two Mrs4 variants, we expressed them in a *C. lusitaniae mrs4*∆ mutant. Both *C. auris* Mrs4^31A^ and Mrs4^31V^ alleles were able to complement *mrs4*∆ for growth with iron chelator and cadmium sensitivity to a similar extent as the functional *C. lusitaniae* Mrs4^REF^ protein (Fig. S8), indicating that both alleles were functional.

**Fig 6:**
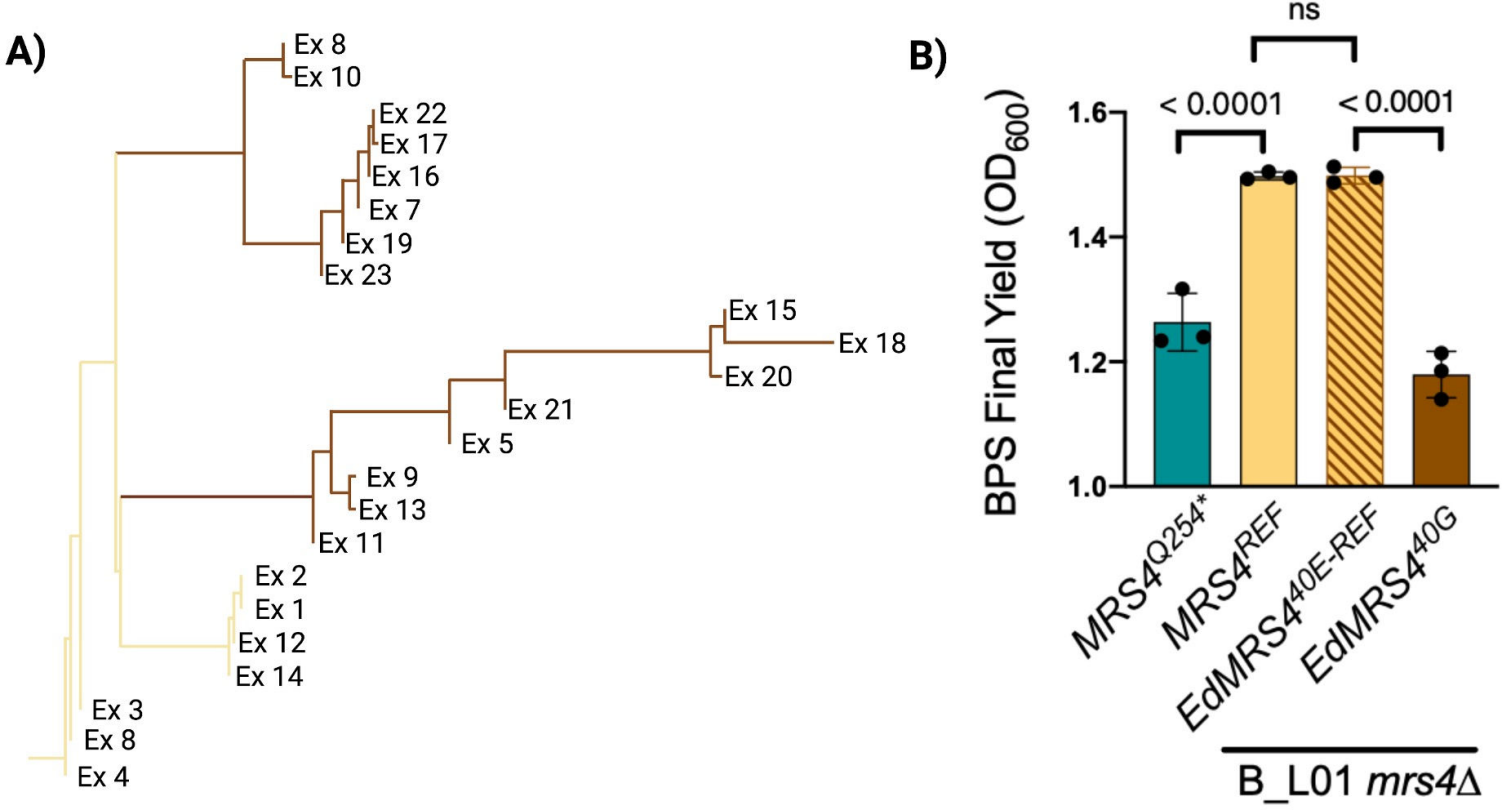
An Mrs4 loss-of-function subpopulation also emerged in *E. dermatitidis* during a chronic CF lung infection. (A) Two alleles of *MRS4* were found in *E. dermatitidis* isolates from a single chronic CF lung infection. Of the 23 isolates sequenced, 7 genomes encoded the reference Mrs440E (*E.d. REF*), which is identical to previously sequenced *E. dermatitidis* strains, and 16 isolates encoded an Mrs4^40G^ variant (*E40G*). (B) *C. lusitaniae* B_L01 *mrs4*∆ strains complemented with the two *E. dermatitidis MRS4* alleles grown in YPD with 80 µM BPS for 24 h. *C. lusitaniae* B_L01 *mrs4*∆ strains expressing functional *MRS4^REF^* or MRS4^Q254*^ were included for comparison. Indicated *P*-value are from a one-way ANOVA with Tukey’s *post hoc* correction. ns, not significant.

The second analysis of Mrs4 variants was in the black yeast *E*. *dermatitidis*. We had previously identified a CF infection predominated by *E. dermatitidis*. We performed whole-genome sequencing of 23 isolates and found a subpopulation of isolates with a non-synonymous SNP in the *MRS4* (Fig. 6A) (33). Subsequent Sanger sequencing confirmed that seven of the 23 isolates carried an *MRS4* ortholog with a sequence identical to the *E. dermatitidis* type strain, NIH8656 (referred to here as encoding Mrs4^40E-REF^). The other 16 sequenced isolates had a variant *MRS4* with an E40G substitution. To characterize the *MRS4* alleles in these *E. dermatitidis* isolates, both were synthesized and heterologously expressed in *C. lusitaniae mrs4*∆ and assessed for function relative to the *C. lusitaniae MRS4* alleles. The *E. dermatitidis* alleles (*EdMRS4^40E-REF^* and *EdMRS4^40G^*) were codon optimized for *Candida* spp. and the spliced introns were removed. The *EdMRS4* alleles were introduced at the native *MRS4* site and expressed under the control of the *C. lusitaniae MRS4* promoter.

Complementation of the *mrs4*∆ strain with Mrs4^40E-REF^ fully complemented the *mrs4*∆ mutant to the levels of *C. lusitaniae MRS4*^REF^. In the presence of the BPS iron chelator, the *mrs4*∆ strain with the *E. dermatitidis* Mrs4^40G^ variant had significantly reduced growth compared to a strain with *E. dermatitidis* Mrs4^40E^, which fully restored the Mrs4 function to levels observed for the *C. lusitaniae* reference gene (Fig. 6B). This indicates that a Mrs4 LOF mutation also arose in a population of *E. dermatitidis* during a CF lung infection. The repeated occurrence of *MRS4* mutations in a variety of fungal CF infections strongly suggests a selective benefit for *MRS4* LOF mutations in the chronically infected CF lung.

## DISCUSSION

In this work, we showed the repeated loss of Mrs4 activity across two fungal species, *C. lusitaniae* and *E. dermatitidis*, in the context of chronic CF lung infections. Each acquired non-synonymous mutations that resulted in reduced or loss of Mrs4 function, which led to defects in iron import into the mitochondrial inner lumen and increased expression and activity of iron-acquiring pathways. The selection for *MRS4* LOF mutations in chronic lung infection may inform future studies on the mechanism of fungal persistence within the host and resistance to therapeutic strategies. The emergence of Mrs4 LOF in two diverged species of Ascomycota, both environmental fungi which colonized chronic CF lung infections, highlights the possibility that mutations in *MRS4* may be important for the shift to commensal-colonizing yeast. Work by Kim et al. (4) found *C. albicans NRG1* LOF mutations in isolates from different individuals with CF suggesting that inactivating mutations in this locus increased fitness. Interestingly, mutants in Nrg1 have increased expression of iron uptake genes as well (34).

In other species, mutation of Mrs4 impairs the mitochondrial synthesis of Fe-S clusters, the synthesis of which is capable of modulating the iron-starvation response through their insertion into low iron transcriptional regulators such as Aft1/2, or modulate activity of low iron response transcription factors Hap43, Sef1, and Sfu1 in ways that are still not understood fully (14, 15, 35). In this manner, Mrs4 mutation broadly promotes the induction of iron-acquisition pathways even in iron-replete conditions as we observed (Fig. 4D). Thus, *MRS4* mutations may be a key mechanism for a simultaneous increase in activity of multiple regulators that cannot be achieved through the mutation of other genes in the pathway. For example, inactivating mutations in genes encoding Sfu1, the transcriptional repressor of iron uptake, or Yfh1, the iron-sulfur exporter, may only activate a subset of pathways or have other pleiotropic effects on the cell. Unlike LOF in Hap43 or gain-of-function mutations in the Sef1 or Hap43 iron transcription factors, which we would predict would result in constitutive derepression of iron uptake, the *MRS4* mutation maintains the ability to regulate the expression of genes involved in iron in response to extracellular iron levels. We expect that moderation of the iron response, which is expressed to a greater degree in Mrs4 LOF than the *MRS4^REF^* strain, is achieved when intracellular iron levels become sufficient for low-affinity iron transporters to compensate for loss of Mrs4 function. Enhanced iron acquistion into the cell with decreased transport into the mitochondria may prevent ROS formation via mitochondrial iron while allowing higher iron levels to be available for enzymes in the cytosol. The increased accumulation of cellular iron may also aid cells during fluctuations in iron availability, such as what might occur in the lung environment that experiences cycles of increased inflammation associated with disease exacerbations and inflammation resolution.

In chronic infections, such as those in the CF lung, the host restricts the availability of essential nutrients such as iron via nutritional immunity (36). Host proteins such as lactoferrin, transferrin, and calprotectin sequester iron, making it less accessible to pathogens (37). Enhanced expression of siderophore acquisition pathways (e.g., Sit1 ortholog CLUG_01760) and surface ferric reductases (e.g., orthologs encoded by CLUG_02348 and CLUG_04344*)* by MRS4^Q254*^ likely provides a significant advantage in acquiring iron from iron-sequestering molecules. *Candida* species also employ a three-factor system of CFEM (common fungal extracellular membrane) proteins for the acquisition of heme-bound iron, which represents approximately 80% of iron within the body (38, 39). The secreted factor Csa2 binds and ferries heme to the cell surface, where it is brought into the cell by Pga7 and Rbt5. Orthologs of these genes (CLUG_4093, CLUG_4096, and CLUG_4097) were at significantly higher levels in MRS4^Q254^^*^ than in *MRS4^REF^* (Fig. 4C and D) in both control and iron-chelated conditions. A preference for heme as the most biologically available source of iron is also observed in chronic bacterial infections, where evidence suggests that heme utilization is selected for and required for virulence (40, 41). Mutations in *MRS4* may also be an important factor in competing with other microbes for iron in its biologically available forms. Mrs4 loss-of-function in chronic conditions is an interesting contrast to the importance of Mrs4 function in systemic fungal infections (19).

Decreased Mrs4 activity may allow cells to accumulate high levels of iron without jeopardizing mitochondrial function. Iron is highly regulated by all cells due to its reactive properties. *MRS4* mutation may not only enhance iron uptake but also limit iron concentrations in mitochondria, which protects mitochondria from damage. Reduced iron uptake by mitochondria may be key for the accumulation of total cellular iron.

One unique feature of the three CF *C. lusitaniae* infections was that there were no detectable bacterial pathogens at the time of the BAL sample collection. When other *Candida* species are detected in CF, the fungi are usually part of a mixed bacterial-fungal infection. The reason for these intriguing differences is not known and a current area of study. Here, we showed that when compared to *C. albicans* SC5314, *C. lusitaniae* produced significantly less ethanol and more acetate as fermentation products. Ethanol stimulates biofilm formation and virulence factor production in CF bacterial pathogens (42, 43), and thus, metabolic differences between fungi may be a factor that differentiates *Candida* species in their interactions with other microbes. Lastly, *C. albicans* and *C. lusitaniae* differ in the degree to which they stimulate macrophages and thus differences in immune response may play an important role (44). Future studies will determine how different species persist in chronic infections, the effects of genetic changes that are repeatedly under selection, and whether there are common themes across different pathogens. Chronic infection by microbes that are not also human commensals, such as *C. lusitaniae* and *E. dermatitidis*, provides an opportunity to study initial adaptations to the host environment and factors that are most critical for survival and persistence.

## MATERIALS AND METHODS

### Clinical isolate collection from respiratory samples

Clinical isolates were acquired from sputum and bronchoalveolar lavage (BAL) fluid samples that were plated on YPD (1% yeast extract, 2% peptone, 2% glucose, 1.5% agar) containing gentamycin, blood agar, or CHROMagar Candida media then restruck on YPD to obtain single isolates which were then saved in 25% glycerol. *C. lusitaniae* clinical isolates were obtained in accordance with the study protocol approved by Dartmouth Health Institutional Review Board (#22781) using methods described in reference (5).

### DNA isolation, genome sequence analysis, and variant calling

Genomic DNA was extracted from cultures grown in YPD (2% peptone, 1% yeast extract, and 2% glucose) for ~16 h; extractions were performed using the MasterPure yeast DNA purification kit (LGC Biosearch Technologies, Beverly, MA, USA) . Genomic libraries, for single and pooled isolate DNA, were prepared using the KAPA HyperPrep Kit and sequenced using paired-end 150 bp reads on the Illumina NextSeq500 platform, to a depth of 100–150× coverage per sample as described in Demers et al. (5). The pipeline for genome analyses is available in a github repository (https://github.com/stajichlab/PopGenomics_Clusitaniae; doi: 10.5281/zenodo.7800401). The short read sequences were aligned to a modified version (5) of the *Candida lusitaniae* ATCC 42720 genome (45). The ATCC 42720 genome was altered to remove mitochondrial fragments inserted into the nuclear assembly, and the mitochondrial contig (Supercontig_9) was replaced by a complete mitochondrial genome from strain *C. lusitaniae* CBS 6936 (NC_022161.1). The following regions were masked out due to unusually high coverage and likely mitochondrial origin: Supercontig_1.2:1869020–90 1869184,1664421–1664580; Supercontig_1.3:1076192–1076578,1324802- 1324956,1353096–1353260; Supercontig_1.6:126390–126604; Supercontig_1.8:29199–92 29370. Alignments were made using bwa (0.7.17-r1188) (46) and stored as a sorted, aligned read CRAM file with Picard (2.14.1, http://broadinstitute.github.io/picard/) to assign read groups and mark duplicate reads (script 01_align.sh). CRAM files were processed to realign reads using GATK’s RealignerTargetCreator v4.1.8.1 and IndelRealigner following best practices of GATK (47). Each realigned CRAM file was processed with GATK’s HaplotypeCaller (script 02_call_gvcf.sh) followed by joint calling of variants on each Chromosome using GATK’s GenotypeGVCF method script 03_jointGVCF_call_slice.sh). This step also removed low-quality variant positions: low-quality SNPs were filtered based on mapping quality (score <40), quality by depth (<2 reads), Strand Odds Ratio (SOR >4.0), Fisher strand bias (>200), and Read Position Rank Sum Test (≤20). These files were combined to produce a single variant call format (VCF) file of the identified variants to produce list of high-quality polymorphisms (script 04_combine_vcf.sh). The quality-filtered VCF file containing only variants among the clinical isolates was categorized by SnpEff (5.1) (48) and the ATCC 42720 gene annotation. Genome assemblies of the strains was performed with SPAdes (v3.12.0) (48) after trimming and adaptor cleanup of the reads was performed with AdapatorRemoval (v2.0) (49) and quality trimming with sickle (v1.33) (50). *De novo* assemblies were further screened for vector contamination with vecscreen step of AAFTF v0.3.1 (https://github.com/stajichlab/AAFTF) (doi: 10.5281/zenodo.1620526). The metadata for the strains corresponding to the genome sequences for isolates from these three subjects will be described in a Microbiology Resource Announcement, and are included in BioProject #PRJNA948351. The raw sequence reads for whole-genome sequencing of subjects A, B, and C isolates have been deposited into NCBI sequence read archive under BioProject #PRJNA948351. Details for the isolates and isolate pools from different regions of the lung for subject A are described in reference (5). The analysis of *MRS4* sequences from environmental *C. lusitaniae* strains (Table S3) and clinical isolates obtained from subsequently obtained sputum or stool sample was performed by amplifying *MRS4* using primers DRM031 and DRM032 and sequenced using these primers along with primer ED157 which binds within the *MRS4* sequence (see Table S4 for primer sequences).

### Strains and mutant construction

Fungal strains and plasmids used in this study are listed in Table S3. Fungi were maintained on YPD medium. CRISPR-Cas9 knockout of *MRS4* from clinical isolates was performed using previously described methods (51). For complementation of the reference *MRS4* allele, 5´ UTR and coding region were amplified from ATCC 42740 and assembled into a complementation plasmid with a marker encoding hygromycin (HYG) resistance and 3´ UTR using yeast recombination cloning (52). The complementation plasmid was digested with NotI and KasI resulting in a ~3,500 bp fragment which was transformed into *mrs4*∆ derivatives of representative isolates along with Cas9 and a crRNA targeting the *NAT* marker.

#### Analysis of *Candida auris MRS4* alleles

Clade I (strain B8441), Clade II (B11220), Clade III (B11221), and Clade IV (B11245) were used in the MRS4 sequence comparisons of *C. auris* isolates. *C. auris MRS4* was amplified from one of two isolates from the CDC Antibiotic Resistance Isolate bank: AR bank #0382 of Clade I (Biosample Accession # SAMN18754596), and AR bank #0383 of Clade III (Biosample Accession # SAMN05379609). *C. auris MRS4* alleles were amplified using primers with 20 base pairs of overlap with *C. lusitaniae MRS4* 5´ UTR and the HYG resistance cassette. *E. dermatitidis MRS4* alleles were synthesized *de novo* by Genscript with the omission of introns, and 20 base pairs of overlap with *C. lusitaniae MRS4* 5´ UTR and the HYG resistance cassette. These sequences were then reintroduced into the native locus by complementation cassette by restriction digest, replacing the reference *MRS4* allele with the heterologous sequences. Complementation of heterologous sequences was then performed by the same method as complementation of the reference allele. Primers are listed in Table S4.

#### Growth assays

Unless otherwise stated, strains were grown as 5 mL cultures in YPD overnight (~16 h), and exponential growth aliquots were washed three times and subcultured into experimental medium. For spot titer growth comparisons, cultures were diluted to 1 OD in diH_2_O, diluted 1:10 serially, and spotted in 5 µL volumes on plates. For growth assays in 96-well plates, a starting concentration of 0.005 OD in YPD was used and stated concentrations of BPS, cobalt chloride, or cadmium chloride were added from stock solutions in water. BPS (Sigma, St. Louis MO, USA, CAS# 52746–49-3), cobalt chloride (Sigma, St. Louis MO, USA, CAS# 7791–13-1), and cadmium chloride (Sigma CAS# 654054–66-7) stocks were 100 mM, 100 mM, and 100 µM, respectively. Final yield was measured by OD600 at 24 h post-inoculation. The BPS, CoCl_2_, and CdCl_2_ concentrations capable of partial inhibition of growth of the *MRS4^REF^* containing strain were determined for each medium empirically. For H_2_O_2_ sensitivity, fresh aliquots of 9.8 M H_2_O_2_ were diluted into YPD at the time of inoculation, and growth was measured over the course of 24 h as previously described. Biolog assays were conducted by suspending 0.01 OD of each strain in YNB, and aliquoting 200 µL of culture into 192 wells of two proprietary Biolog plates PM1 and PM2A with a diverse array of carbon sources. Growth was measured by OD600 over the course of 48 h, and the final yield is represented in Supplementary Dataset 1.

#### TTC analysis of surface iron reductase activity

After 24-h growth on the indicated medium, a 10 mL solution containing 0.5 mg/mL or 1 mg/mL tetrazolium chloride, 1% molten agar, and 10 mM FeCl_3_ chloride (from 100 mM stock made fresh), if indicated, was carefully pipetted using a 10 mL serological pipette to cover the entirety of the plate. Plates were incubated for the specified time (10 min to 1 h) prior to imaging.

#### Transcriptome analysis of the effects *MRS4* mutation

For RNA isolation, isolates were sub-cultured from overnight cultures into fresh YPD and grown for 6 h which corresponds to cultures in mid-exponential growth phase. Cultures were sub-cultured into six replicate 5 mL cultures of each strain which were incubated at 37°C on a roller drum. After 5 h of growth, three replicate cultures of each strain were dosed with BPS to a final concentration of 80 µM, while the other cultures received water only. Samples were spun down in 15 mL conical tubes, snap-frozen with ethanol and dry ice, and stored for at least 1 h at −80˚C. RNA extraction was performed using MasterPure Yeast RNA Purification kit protocol (Epicentre) according to manufacturer instructions. RNA was submitted to MiGS for RNA-seq analysis. DEseq was used for normalization and differential gene analysis of raw counts provided by MiGS. Heatmaps depict the Z-scores of differentially expressed genes with a log_2_ fold-change of ≥1, and *P*-value <0.05. RNA-seq data have been submitted to the SRA database, BioProject #PRJNA831802, experiments #SRX19760020-19660043.

#### qRT-PCR analysis

Culture growth and RNA extraction was performed as described above for the RNA-seq analyses. RNA was DNAse treated with the Turbo DNA-free Kit (Invitrogen, Waltham, MA, USA). cDNA was synthesized from 500 ng DNAse-treated RNA using the RevertAid H Minus First Strand cDNA Synthesis Kit (Thermo Scientific, Waltham, MA, USA), following the manufacturer’s instructions for random hexamer primer (IDT, Coralville, IA, USA) and GC rich template. qRT-PCR was performed on a CFX96 Real-Time System (Bio-Rad, Hercules, CA, USA), using SsoFast Evergreen Supermix (Bio-Rad, Hercules, CA, USA) with the primers listed in Table S4. Thermocycler conditions were as follows: 95°C for 30 s, 40 cycles of 95°C for 5 s, 65°C for 3 s, and 95°C for 5 s. Transcripts were normalized to *ACT1* expression.

#### Intracellular iron quantification

Cells from overnight cultures were subcultured into YPD and grown for 5 h before the addition of 80 µM of BPS iron chelator. Samples were taken before adding chelator, and 1 h after iron restriction. Samples were spun down in pre-weighed Eppendorf tubes, washed, and pellets were dried in a vacuum centrifuge for 3 h. Final dry weight was calculated for each pellet, and each was digested with 100 µL of 70% HNO_3_. After overnight digestion, samples were heated to 90°C to ensure complete digestion. After dilution with 3.9 mL of diH_2_O, samples were submitted to the Dartmouth Trace Metal Core for ICP-MS analysis of iron content.

#### HPLC supernatant analysis

Strains grown in overnight cultures were washed three times in dH_2_O and subcultured in triplicate into YNB minimal media supplemented with 100 mM glucose at a final OD of 0.01. Cultures were allowed to grow for 6 h at 37°C, then centrifuged at 13,200 rpm for 5 min for the separation of insoluble solids and collection of supernatant. Four blank media samples of YNB were also prepared with known concentrations of added carbon sources. One blank was supplemented with 100 mM glucose, 5 mM sodium acetate, 100 mM ethanol, 5 mM sodium citrate, 500 µM sodium lactate, 5 mM sodium succinate, 200 µM sodium pyruvate, and 100 mM glycerol, with two other blanks containing 1:10 and 1:100 dilutions of these carbon sources for the creation of a standard curve within a linear range. The final blank was prepared without any additional carbon sources. For each sample, 400 µL of supernatant was centrifuged at 10,000 rpm for 2.5 min through Corning nonsterile nylon 0.22 X-spin filters (#8169), then 20 µL of 10% sulfuric acid as added. Samples were transferred to 2 mL polypropylene snap top microvials for HPLC analysis. Samples were analyzed for levels of various sugars and organic acids utilizing a Shimadzu HPLC (LC-2030) with Biorad Aminex HPX-87H column, LC-20AD pump system, SPD-20AV detector, SIL20AC autosampler, and CTO-20AC column oven.

#### Statistical analysis

All data were analyzed using Graph Pad Prism 8. The data represent the mean standard deviation of at least three independent experiments with three technical replicates unless stated otherwise. Comparisons were made using a two-tailed, unpaired Student’s *t*-test or ANOVA as indicated. One-way ANOVA tests were performed across multiple samples with Tukey’s multiple comparison test for unpaired analyses.

## Data Availability

Names of custom codes used for analysis are indicated where appropriate in the above methods. All codes and sequences are available in the indicated github repositories: analysis pipeline and scripts for whole genome genotyping and phylogeny analysis are available at GitHub . These are archived with Zenodo under doi 7800401. Analysis pipeline for RNA-seq data is available at GitHub.

## SUPPLEMENTAL MATERIAL

Data Set S1 mbio.01171-23-s0001.xlsx Biolog carbon utilization phenotype microarray plates.

Data Set S2 mbio.01171-23-s0002.xlsx Gene expression differences.

Supplemental Figures and Tables mbio.01171-23-s0003.pdf Figures S1-S8 and Tables S1-S4.

ASM does not own the copyrights to Supplemental Material that may be linked to, or accessed through, an article. The authors have granted ASM a non-exclusive, world-wide license to publish the Supplemental Material files. Please contact the corresponding author directly for reuse.
