# Supplementary material for: Mrs4 loss of function in fungi during adaptation to the cystic fibrosis lung
Source: mBio. 2023 Jul 11;14(4):e01171-23. doi: 10.1128/mbio.01171-23 (PMC10470810; doi:10.1128/mbio.01171-23)
Supplement: Supplemental Figures and Tables — Figures S1-S8 and Tables S1-S4. [file mbio.01171-23-s0003.pdf]

```
CLUSTAL O(1.2.4) multiple sequence alignment


UT8656          ----MTLMNSPD---AVPGEEFDYEALPPNYGLGHNMLAGAFAGIAEHTVMYPVDLMKTR     53
S288C           MVENSSSNNSTRPIPAIPMDLPDYEALPTHAPLYHQLIAGAFAGIMEHSVMFPIDALKTR     60
SC5314          ------MEHQLQFLPKDS-VEIDYEALPDDASLAAHLTAGALAGIMEHTVMFPIDSIKTR     53
ATCC42740       ------MADHPHQLGADT-LEVDYEALPEDASIIAHLSAGAFAGIMEHTVMYPIDSIKTR     53
B11220          ------MADHPHMLS-EP-IEIDYEALPEDASIAAHLSAGAFAGIMEHTVMFPIDSIKTR     52
                        .             ****** .  :  :: ***:*** **:**:*:* :***

UT8656          MQIINPSAGGLYTGLSHAVSTIYRLEGLRTLWRGVTSVIVGAGPAHAVYFGTYEMVKELA     113
S288C           IQSANAKS-LSAKNMLSQISHISTSEGTLALWKGVQSVILGAGPAHAVYFGTYEFCKKNL     119
SC5314          MQMNLSNS-EISRGLLKSLSKISSTEGFYALWKGVSSVVLGAGPAHAIYFSVFESTKTFL     112
ATCC42740       MQIIASGQ-ASSRSVISSISRISSSEGAYALWRGVSSVVMGAGPAHAVYFSVFEATKTML     112
B11220          MQMIASGQ-PLSKSVIGSISKISSSEGAYALWRGVSSVVLGAGPAHAVYFSVFEATKTML     111
                :*           .:   :* *   **  :**:** **::*******:**..:*  *

UT8656          GTNST---------DGKHHPFAAAASGAAATITSDALMNPFDVIKQRMQVHGST------     158
S288C           IDSSD---------TQTHHPFKTAISGACATTASDALMNPFDTIKQRIQLNTS-------     163
SC5314          VNRLTNSPHSNRIVTDENHPLIASCAGITGTTASDALMTPFDMLKQRMQANAAYQDGK--     170
ATCC42740       VNRLTNSS-STKIVTDESHPLIASGAGIAATIASDALMTPFDVLKQRMQVGNEAKA----     167
B11220          VNRLTNSR-STKIVTDESHPLIASGAGIAATVASDALMTPFDVLKQRMQLGTSTADSKLT     170
                                  **: :: :*  .* :*****.*** :***:*

UT8656          ---YRSLTHCAREIFRTEGFSAFYVSYPTTLCMTVPFTATQFMAYESLSTIMNPKKEYDP     215
S288C           ----ASVWQTTKQIYQSEGLAAFYYSYPTTLVMNIPFAAFNFVIYESSTKFLNPSNEYNP     219
SC5314          -STSVRLFKLASDIYKAEGLSAFYISYPTTLLTNIPFAALNFGFYEYSSSLLNPSHVYNP     229
ATCC42740       --SSVKLLSTALSIYKTEGASAFFISYPTTLFTNIPFAALNFGFYEYCSSLLNPENTYNP     225
B11220          QSKSARLVKTAAQIYSKEGLSAFFISYPTTLFTNIPFAALNFGFYEYSSKLLNPENKYNP     230
                      :   : .*:  ** :**: ******  .:**:* :*  **  :.::**.: *:*

UT8656          ITHCVAGGLAGAFAAGITTPLDVIKTLLQTRGLSQ--KDEIRNVRGLFHAASIIKREFGW     273
S288C           LIHCLCGSISGSTCAAITTPLDCIKTVLQIRGSQTVSLEIMRKADTFSKAASAIYQVYGW     279
SC5314          YLHCVSGGIAGGIAAALTTPFDCIKTVLQTKGISQ--NQNFRHVTGFKSAAVALLKQEGA     287
ATCC42740       YYHCVSGGIAGGIAAALTNPFDCIKTALQTRGIST--NESLRNVNGFSSAARALYRHGGF     283
B11220          YYHCISGGIAGGIAAALTNPLDCIKTALQTRGVSQ--NESLRTINGFVSAAKALYKQGGA     288
                  **:.*.::*. .*.:*.*:* *** ** :* .    : :*    :  **  : :  *

UT8656          SGFMRGWRPRIISTMPSTAICWSSYEMAKAYFKRTLREEHEAQMSKL  320
S288C           KGFWRGWKPRIVANMPATAISWTAYECAKHFLMTY------------  314
SC5314          KAFWKGLKPRVIFNIPSTAISWTAYEMCKEVLIRGKGL---------  325
ATCC42740       GAFMRGLKPRIIFNVPSTAISWTAYEMAKEVLLRD------------  318
B11220          GAFTRGLKPRIIFNVPSTAISWTAYEMAKEVLLRD------------  323
                 .* :* :**:: .:*:***.*::** .*  :
```

Figure S1. Non-synonymous SNPs occur in highly conserved regions of Mrs4 orthologs. Clustal Omega (Sievers et al., 2011) generated alignment of representative *MRS4* peptide sequences from *E. dermatitidis* (UT6856*), S. cerevisiae (*S288C*), C. albicans* (SC5314), *C. lusitaniae* (ATCC 42740)*,* and *C. auris* (B11220)*.* Missense and nonsense mutations with functional effects are highlighted: *C. lusitaniae* G138 (light purple), A147 (dark purple), A235 (red), and Q254 (cyan). The residue that corresponds to *E. dermatiditis* E40 (brown) is also shown. For full citation, see Supplemental References.

Synonymous SNPs

	DH2383	ATCC	DH3115	DH3116	DH3117	DH3118	DH3119	DH3120	DH3121	DH3122
DH2383	0									
ATCC	0	0								
DH3115	1	1	0							
DH3116	1	1	0	0						
DH3117	0	0	1	1	0					
DH3118	1	1	0	0	1	0				
DH3119	5	5	6	6	5	6	0			
DH3120	5	5	6	6	5	6	0	0		
DH3121	6	6	6	7	6	6	4	4	0	
DH3122	6	6	7	7	7	7	5	5	5	0

Figure S2. The *MRS4* sequences of environmental, reference, and acute infection isolates of *C. lusitaniae* differ by only a small number of synonymous SNPs. Comparison of synonymous SNPs in the *MRS4* sequences encoding the "reference" amino acid sequence. *MRS4* alleles of *C. lusitaniae* clinical strains (DH2383 and ATCC 42740) and environmental isolates (see Table S3) differ only by the number of synonymous mutations indicated in the matrix.

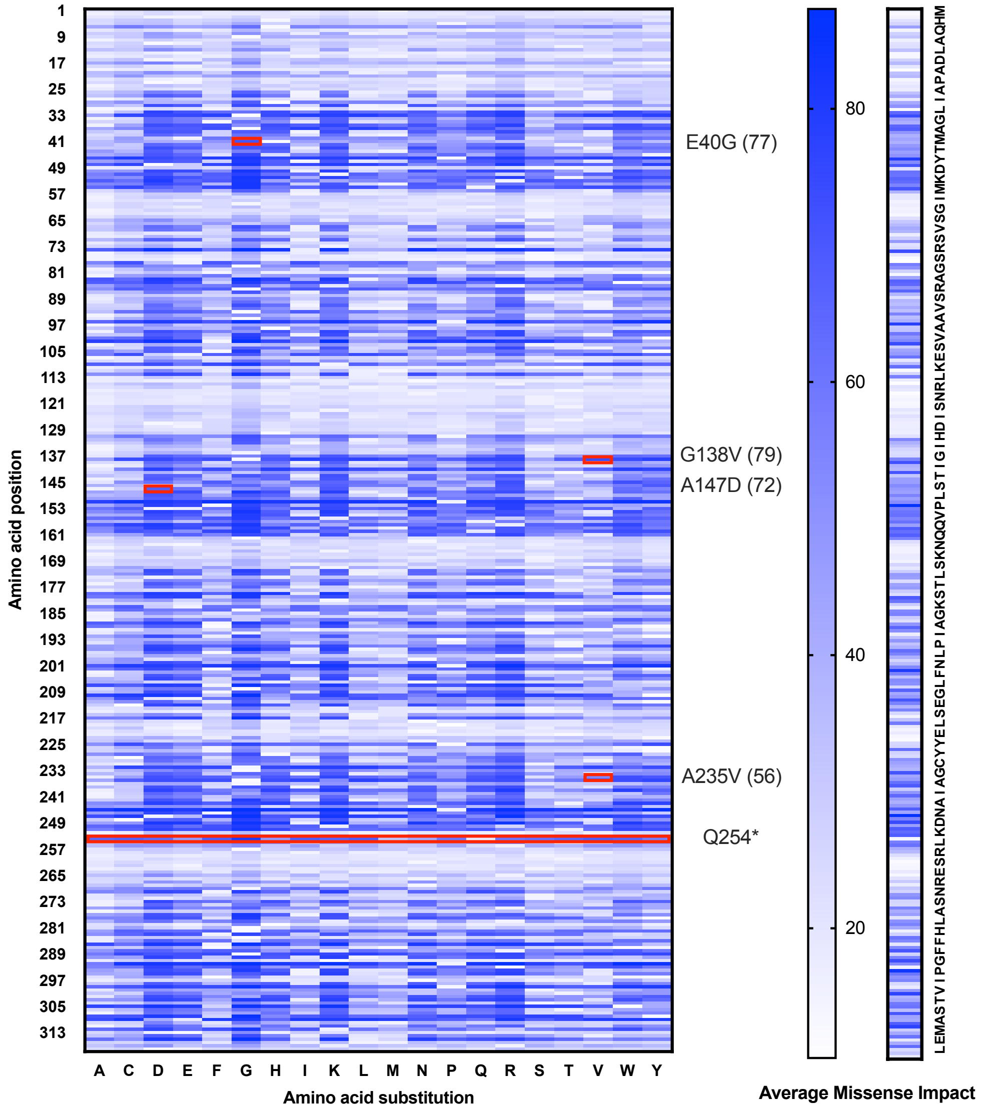

Figure S3. Non-synonymous SNPs occur in predicted high-severity impact regions. SuSPect (Yates, Filippis, Kelley, & Sternberg, 2014) mutational analysis heatmaps indicate that the predicted severity of each possible particular amino acid substitution at a given position and the average impact of all possible amino acid substitutions on a scale from 0 (least severe) to 100 (most severe). Higher areas of impact coincide with predicted transmembrane alpha helix regions (see **Fig. 1A**). *C. lusitaniae MRS4* mutations are highlighted in red with their predicted impact score in parentheses (except for the Q254* mutation). The predicted effects of the *E. dermatiditis MRS4* substitution, which leads to an E40G substitution, is also shown. For full citation, see Supplemental References.

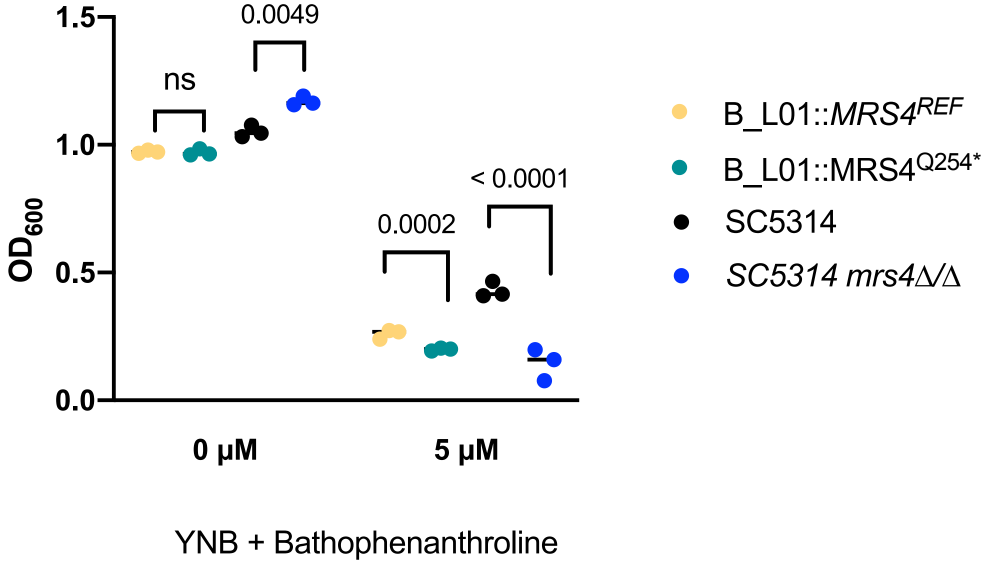

Figure S4. Restriction of *mrs4* growth by chelator in defined minimal media. B_L01 *mrs4*Δ complemented with *MRS4*^{REF} and *MRS4*^{Q254*} alleles, and SC5314 parent and *mrs4*Δ/Δ derivative were inoculated into Yeast Nitrogen Base, 2% glucose with and without 5 µM bathophenanthroline, sensitivity to low iron is observed in *MRS4* defective or knockout strains in *C. lusitaniae* and *C. albicans* respectively. Indicated p-values are from Student's T-Test with Holms-Sidak correction.

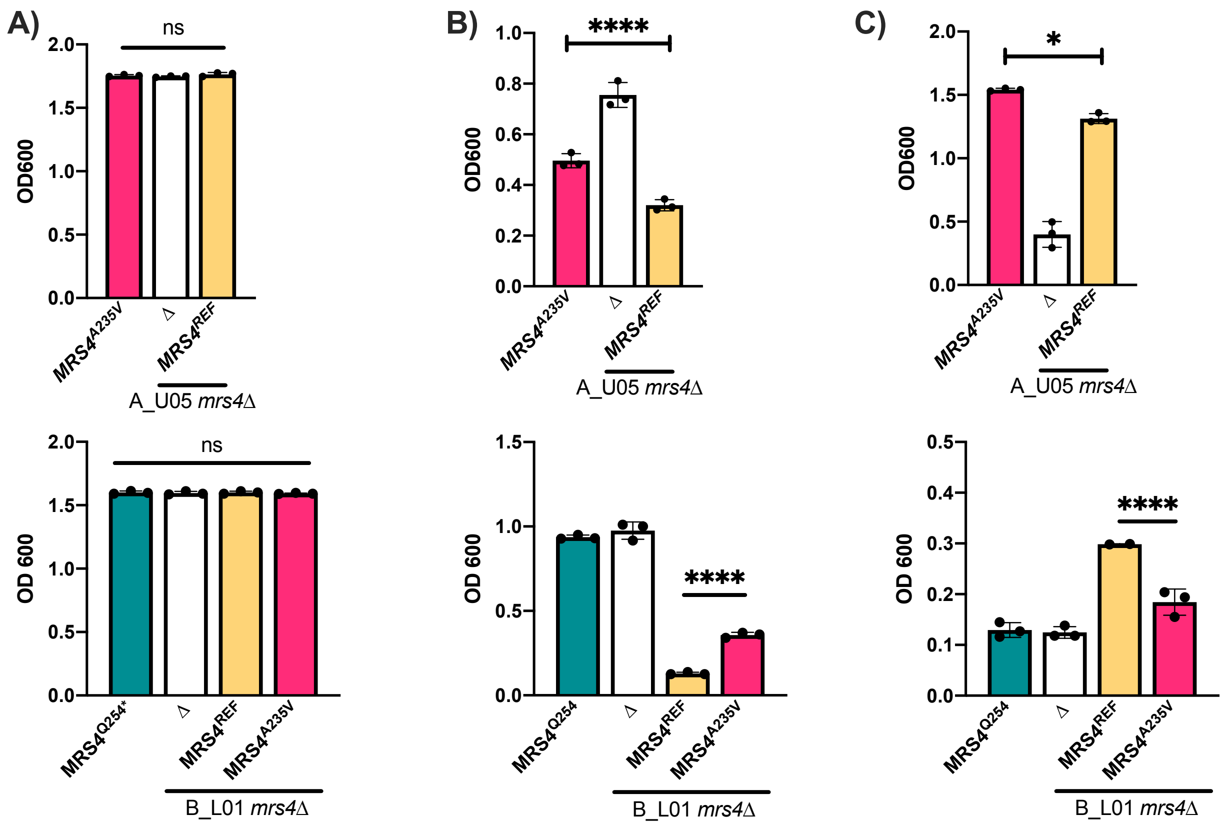

Figure S5. The A_U10D strain background masks the Mrs4-dependent phenotype of cadmium sensitivity. A) Effects of *MRS4^{A235V}* on growth in YPD in the A_U05 (top) and B_L01 (bottom) backgrounds. The *mrs4*Δ, and *mrs4*Δ+*MRS4^{REF}* are shown for both strains and *mrs4*Δ+*MRS4^{A235V}* in the B10D background. Growth for the same strains as in panel A in 2.5 mM $CoCl_3$ **B)** and 12.5 mM $CdCl_2$ **C)** are shown. Data shown in panel C (top) is also shown as part of Fig. 2F.

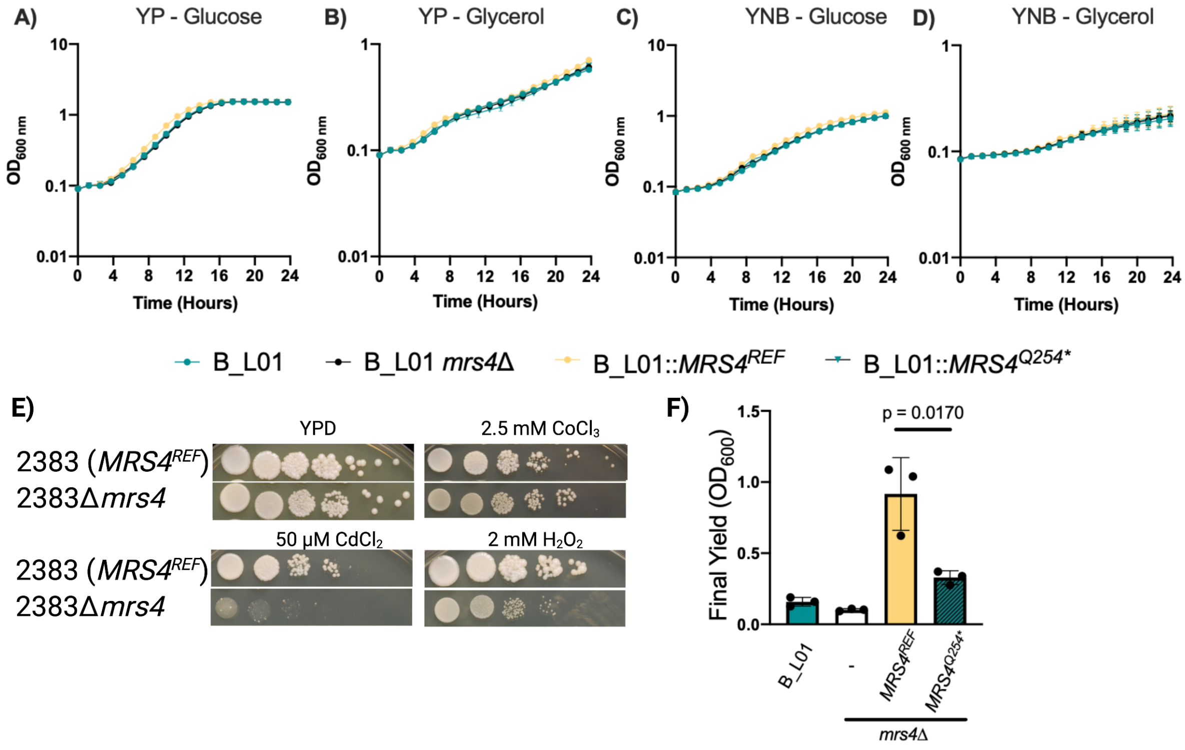

Figure S6. Deletion of *MRS4* does not impact carbon source dependent growth, but confers sensitivity to ROS. Cells from exponential phase cultures of B_L01, its *mrs4*Δ derivative, and *mrs4*Δ mutants complemented with *MRS4*^{Q254*} or the *MRS4*^{REF} were inoculated into a 96-well plate and grown for 24 h at 37˚ in YP medium supplemented with A) 2% glucose or B) 2% glycerol or in YNB defined medium without amino acids supplemented with C) 2% glucose or D) 2% glycerol. OD_{600} was measured over time using a Synergy Neo2 plate reader. The exponential phase growth rates were not significantly different across the three strains in any of the conditions in three independent experiments. **E)** An *mrs4*Δ strain, constructed in DH2383, which has *MRS4*^{REF} sequence, and the DH2383 parent strain were spotted in a dilution series on YPD plates supplemented with cobalt, cadmium, and hydrogen peroxide as described in the Materials and Methods for 48 h before imaging. **F)** B_L01 parent, *mrs4*Δ*,* and complemented strains with the *MRS4*^{REF} or native *MRS*^{Q254*} allele were grown in a 96-well plate format in YPD containing 1 mM H_2O_2 for 24 h at 37˚. Final yield was measured as absorbance at 600 nm on a Synergy Neo2 plate reader.

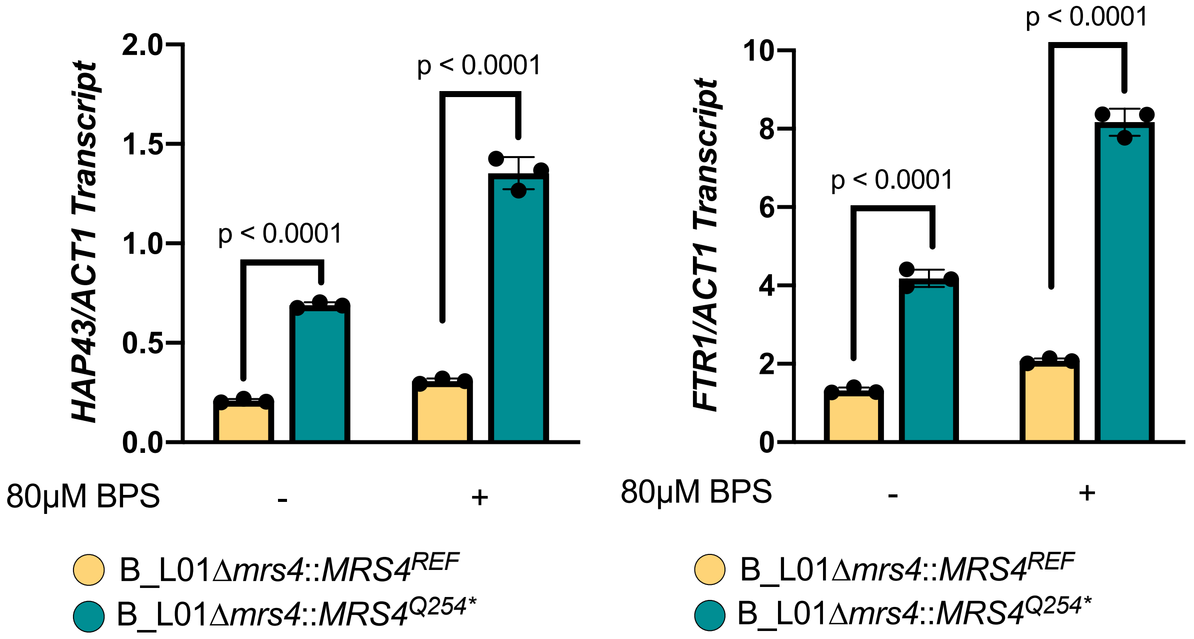

Figure S7. Quantitative RT-PCR analysis of *HAP43* and *FTR1* in the absence and presence of Mrs4 activity in iron-chelated conditions. Transcripts encoding the iron-regulating transcription factor Hap43 and high-affinity iron transporter Ftr1 were measured in B_L01 *mrs4*Δ strains complemented with $MRS4^{REF}$ or native MRS^{Q254*} in cells grown in YPD for 6 h, or in YPD for 5 h followed by 1 h growth after the addition of 80μM BPS.

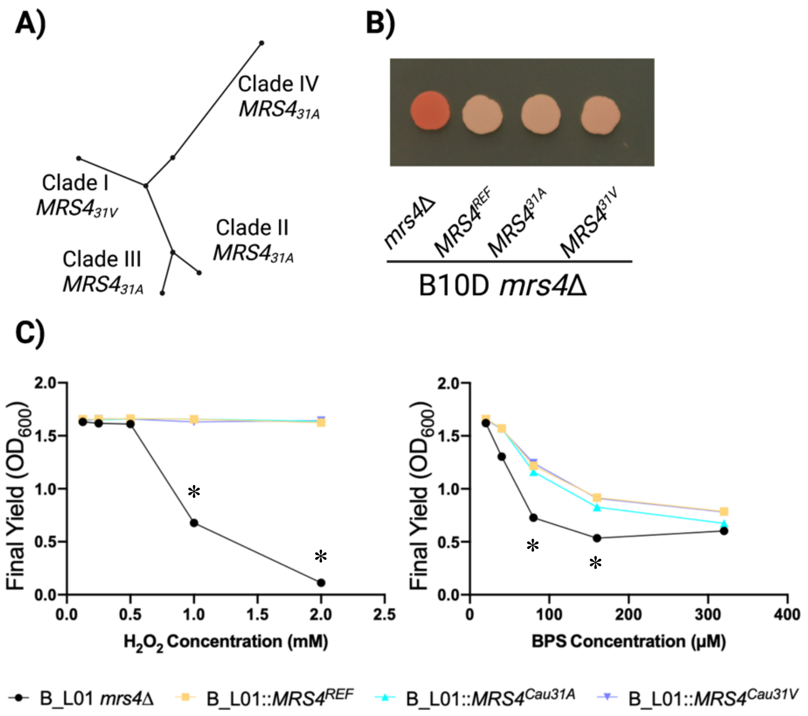

Figure S8. The *C. auris* clade I ortholog of *MRS4* differs from other clades by a single amino acid change that does not affect Mrs4 function. A) *C. auris* clade distribution of *MRS4* alleles, with phylogenetic tree based on (Du et al., 2020). For full citation, see Supplemental References. **B)** TTC reduction phenotypes of *C. auris MRS4* alleles heterologously expressed in the B_L01 background, spotted on YPD plates and grown for 24 h before TTC overlay. **C)** Growth of *C. auris* alleles in BPS and H_2O_2.

Table S1: Loci with heterogeneity in encoded amino acid sequences within more than one CF-associated *C. lusitaniae* population. Whole genome SNP tables were compiled from the sequencing of chronic infection isolates from each individual with CF and compared against each other. Genes which had acquired unfixed mutations (multiple alleles within the population) in more than one population are listed below. *MRS4* is the only gene which had unfixed mutations in all three independent populations, and is not listed here among genes which had unfixed mutations associated with two independent populations. *C. albicans* gene names or their alias are denoted next to their predicted *C. lusitaniae* ortholog, which was determined by Candida Genome Database.

Variable loci in Subj. A and B (4)	***Variable loci in Subj. A and C (7)***	***Variable loci in Subj. B and C (8)***
CLUG_03891	CLUG_03989	CLUG_03981
CLUG_00379	CLUG_01165	CLUG_03274 (ALS4)
CLUG_03468 (RPO21)	CLUG_05802	CLUG_01977
CLUG_02919	CLUG_02244 (MEF1)	CLUG_02910
	CLUG_00052 (NUP145)	CLUG_04108
	CLUG_05353 (SPL1)	CLUG_01201
	CLUG_03194	CLUG_01123 (CBK1)
		CLUG_05462

Table S2: Mrs4 loss of function does not alter secondary carbon metabolite secretion in *C. lusitaniae.* B_L01 derivatives B_L01::*MRS4*^{REF} and B_L01::*MRS4*^{Q254*}, and *C. albicans* SC5314 with single and double knockouts of *MRS4,* were cultured overnight and sub-cultured into YNB minimal media with 100mM glucose as a primary carbon source. Supernatants were collected and analyzed by HPLC for consumption of glucose, and production of major secondary metabolites. Molar carbon ratios were calculated for consumed glucose and citrate to produced acetate, ethanol, and glycerol, relative to the base media.

	C. lusitaniae B_L01		*C. albicans* SC5314	
Substrates (mM)	*MRS4*^{REF}	*MRS4Q254**	*MRS4/ MRS4*	*mrs4* Δ/Δ
Glucose utilized	43.9	27.5	63	59.6
Citrate utilized	2.6	2.37	2.50	1.90
**Average Carbon Consumed**	**279**	**179**	**392**	**369**
Acetate produced	41.4	23.9	3.38	2.56
Ethanol produced	13.4	5.14	24.9	29.3
Glycerol produced	nd	nd	0.08	0.02
**Average Carbon Produced**	**110**	**58.1**	**56.9**	**63.8**
**Carbon produced: Carbon utilized**	**0.39**	**0.32**	**0.15**	**0.17**

Table S3. Strains and plasmids

Strain	Strain background	Marker	Lab #	Source
C. lusitaniae				
ATCC 42740			DH2387	(Butler et al., 2009)[a]
DH2383(RSY284/ CL6936)			DH2383	(Reedy, Floyd, & Heitman, 2009)
*mrs4*Δ	DH2383	NatR	DH4093	This study
A_U05			DH3087	Dartmouth Health
B_L01			DH3703	Dartmouth Health
mrs4	“	NatR	DH4080	This study
mrs4::MRS4^{REF}	“	HygB-R	DH4081	This study
mrs4::MRS4^{Q254*}	“	HygB-R	DH4082	This study
mrs4::MRS4$^{Cau\text{-}31V}$ *(C. auris)*	“	HygB-R	DH4083	This study
mrs4::MRS4$^{Cau\text{-}31A}$ *(C. auris)*	“	HygB-R	DH4084	This study
mrs4::MRS4$^{Ed\text{-}40G}$ *(E. dermatiditis)*	“	HygB-R	DH4085	This study
Mrs4::MRS4$^{Ed\text{-}40E}$ *(E. dermatiditis)*	“	HygB-R	DH4086	This study
A_U05 *mrs4*Δ	A_U05	NatR	DH4087	This study
*mrs4*Δ*::MRS4*^{REF}	A_U05	HygB-R	DH4088	This study
B_L04			DH3706	Dartmouth Health
*mrs4*Δ	B_L04	NatR	DH4089	This study
*mrs4*Δ*::MRS4*^{REF}	B_L04	HygB-R	DH4090	This study
C_M06			DH3708	Dartmouth Health
*mrs4*Δ	C_M06	NatR	DH4091	This study
*mrs4*Δ*::MRS4*^{REF}	C_M06	HygB-R	DH4092	This study
A_S22	Sp2 isolate from Subj. A			
A_S23	“			
A_S24	“			
A_S25	“			
B_S17	Sp4 from subject B			
B_S18	“			
61-4	Environ.		DH3115	PYCC**
71-129	Environ.		DH3116	PYCC**
76-31	Environ.		DH3117	PYCC**
79-1	Environ.		DH3118	PYCC**
80-11	Environ.		DH3119	PYCC**
80-12	Environ.		DH3120	PYCC**

82-606.2	Environ.		DH3121	PYCC**
16-4994.55	Environ.		DH3122	PYCC**
ATCC 42720	Clinical		DH2387	(Butler et al., 2009)
Acute 1A	Acute		DH3769	This study
AR0398	Unknown		DH2785	CDC panel
1H	Clinical, ascending colon, *MRS4*^{A147D}		DH4021	This study
6B	Clinical, transverse colon, *MRS4*^{A147D}		DH4023	This study
6D	Clinical, transverse colon, *MRS4*^{A147D}		DH4025	This study
11B	Clinical, descending colon, *MRS4*^{A147D}		DH4031	This study
11G	Clinical, descending colon, *MRS4*^{A147D}		DH4036	This study
11H	Clinical, descending colon, *MRS4*^{A147D}		DH4037	This study
Candida albicans				
SC5314			DH35	
mrs4	SC5314	NatR	DH4094	This study
*mrs4*Δ/Δ	SC5314	NatR	DH4095	This study
**Plasmids**				
pDRM03	*pMQ30-MRS4REF-HygR*		DH4096	
pDRM06	*pMQ30-MRS4Q254*-HygR*		DH4097	
pDRM10	*pMQ30-MRS4-*^{31A}*(C. auris)*		DH4098	
pDRM13	*pMQ30-MRS3-*^{31V}(*C. auris*)		DH4099	
pDRM14	*pMQ30-MRS440G* (*E. dermatiditis*)*-HygR*		DH4100	
pDRM15	*pMQ30-MRS440E* (*E. dermatiditis*)*-HygR*		DH4101	

[a]See supplement references for full citations.

Table S4. Primers

Primer Label	Sequence	Descriptor
ED149	CTTTGCCTCACATGGGCACTC	CLUG_02526 LF FWD
ED150	gtattctgggcctccatgtcGGTTGGATGGTAGAATCAGAAGAAG	CLUG_02526 LF REV
ED151	gaatgctggtcgctatactgGATTGCGGACATGTGTATTGGTTG	CLUG_02526 RF FWD
ED152	CACTAGATCATGGAGTATGCGG	CLUG_02526 RF REV
ED153	CTTCTTCTGATTCTACCATCCAACCgacatggaggcccagaatac	CLUG_02526 + NAT FWD
ED154	CAACCAATACACATGTCCGCAATCcagtatagcgaccagcattc	CLUG_02526 + NAT REV
ED155	GATATGGGTTGTGGACAATCTCCG	CLUG_02526 NEST FWD
ED156	CTCTTCTAACTCATGCCTGATCTCG	CLUG_02526 NEST REV
ED157	CATCTGCTATCTGTTGCTG	CLUG_02526 Sanger Sequencing
ED179	GATTAAGTTGGGTAACGCCAGGCGCCCTTTGCCTCACATGGGCACTC	CLUG_02526 LF Fwd + pMQ30 overlap
ED180	CAAGCACTATACGACGTCAGGTCTAGAGTAGACAATGTGCCTATACTTG	CLUG_02526 Rev + Hyg overlap
ED181	CAAGTATAGGCACATTGTCTACTCTAGACCTGACGTCGTATAGTGCTTG	Hyg FWD + CLUG_02526 overlap
ED182	GTTTCGTATGCAATGTAATGGATAGAAAGGGCCTCGTGATACGC	Hyg Rev + CLUG_02526 overlap
ED183	GCGTATCACGAGGCCCTTTCTATCCATTACATTGCATACGAAAC	CLUG_02526 RF Fwd + Hyg overlap
ED184	GTATGTTGTGTGGAATTGTGAGGCGGCCGCCTCTTCTAACTCATGCCTGATC	CLUG_02526 RF rev + pMQ30 overlap
DRM019	CTTGTATGAGGCGGTCTTTC	pDRM03 Sequencing
DRM020	CAACTTGGGGCTGACACAC	pDRM03 Sequencing
DRM021	GACAGAGGGTGCCAGTGC	pDRM03 Sequencing
DRM022	CATGTAATAAACGGATACGGCA	pDRM03 Sequencing
DRM023	GATGTATTTACCAACGCCATC	pDRM03 Sequencing
DRM024	CTTTCGAAACATGGATAACGAG	pDRM03 Sequencing
DRM031	ggcattaaagaagggcaagg	*mrs4* External 5' check
DRM032	gactcatctcctggcatgaa	*mrs4* External 3' check
DRM041	GATTAAGTTGGGTAACGCCAATGGCCGACCATCCCCATATG	C. auris MRS4 Fwd + CLUG_02526 LF overlap
DRM042	AAGCACTATACGACGTCAGGCTAGTCTCTCAACAACACCTCCTTGG	C. auris MRS4 Rev + Hyg overlap
DRM045	TCTGATTCTACCATCCAACCATGACTCTAATGAACAGCCCGGATG	E. derm MRS4 Fwd + CLUG_02526 LF overlap
DRM047	AAGCACTATACGACGTCAGGTCAGAGCTTGCTCATTTGAGCC	E. derm MRS4 Rev + Hyg overlap
DRM073	CTTCTGATTCTACCATCCAACC	pDRM014/015 sequencing
DRM074	CCGGTCGCTCACACATTG	pDRM014/015 sequencing
DRM075	CAGAGCTTGCTCATTTGAGCC	pDRM014/015 sequencing

DRM076	CACGGCGATTTGCTGGTC	pDRM014/015 sequencing
DRM106	CCGCGTCAGATGCTTTGATGCAAATTAAAAATAGTTTACGCAAGTC	SNR52-CaMRS4proto linker Rev
DRM107	CATCAAAGCATCTGACGCGGGTTTTAGAGCTAGAAATAGCAAGTTAAA	Calbicans-MRS4 Protolinker-sgRNA scaffold Fwd
DRM108	AGAAGCATAAAGAAACAAGCAATTCGTGGCTCAGACAAGGACACAGATAATACTTAAATTAATAATACAATACTATCATATTTTCCCAGTCACGACGTT	Calbicans-MRS4 linked-NAT KO Cassette Fwd
DRM109	TAGTAATGAAACAAAAACGTTATCTGAAATTATTTACAAATTAGAAAAATAAATACCAAGTAGTATGATGTCTATGAAAAGTGGAATTGTGAGCGGATA	Calbicans-MRS4 linked-NAT KO Cassette Rev
DRM110	GAAGTGTGCGTGTGTGAGC	C. albicans MRS4 KO 5' Check
DRM111	GCGCCCAATACCACAGAAG	C. albicans MRS4 KO 3' Check

Supplement References

Butler, G., Rasmussen, M. D., Lin, M. F., Santos, M. A., Sakthikumar, S., Munro, C. A., . . . Cuomo, C. A. (2009). Evolution of pathogenicity and sexual reproduction in eight *Candida* genomes. *Nature, 459*(7247), 657-662. doi:10.1038/nature08064

Du, H., Bing, J., Hu, T., Ennis, C. L., Nobile, C. J., & Huang, G. (2020). Candida auris: Epidemiology, biology, antifungal resistance, and virulence. *PLoS Pathog, 16*(10), e1008921. doi:10.1371/journal.ppat.1008921

Reedy, J. L., Floyd, A. M., & Heitman, J. (2009). Mechanistic plasticity of sexual reproduction and meiosis in the Candida pathogenic species complex. *Curr Biol, 19*(11), 891-899. doi:10.1016/j.cub.2009.04.058

Sievers, F., Wilm, A., Dineen, D., Gibson, T. J., Karplus, K., Li, W., . . . Higgins, D. G. (2011). Fast, scalable generation of high-quality protein multiple sequence alignments using Clustal Omega. *Mol Syst Biol, 7*, 539. doi:10.1038/msb.2011.75

Yates, C. M., Filippis, I., Kelley, L. A., & Sternberg, M. J. (2014). SuSPect: enhanced prediction of single amino acid variant (SAV) phenotype using network features. *J Mol Biol, 426*(14), 2692-2701. doi:10.1016/j.jmb.2014.04.026
